# Development, validation, and application of a dual-color fluorescent assay for high-throughput screening of anti-chikungunya drugs

**DOI:** 10.1038/s41598-025-16087-1

**Published:** 2025-08-22

**Authors:** Pattadon Sawetpiyakul, Duangpron Peypala, Pathaphon Wiriwithya, Gridsada Phanomchoeng, Tanatorn Khotavivattana, Warintorn Chavasiri, Sittiporn Pattaradilokrat, Siwaporn Boonyasuppayakorn

**Affiliations:** 1 Center of Excellence in Applied Medical Virology, Department of Microbiology, Faculty of Medicine, Chulalongkorn University, Bangkok, 10330 Thailand; 2 Department of Biology, Faculty of Science, Chulalongkorn University, Bangkok, 10330 Thailand; 3 Department of Mechanical Engineering, Faculty of Engineering, Chulalongkorn University, Bangkok, 10330 Thailand; 4 Center of Excellence in Natural Product, Department of Chemistry, Faculty of Science, Chulalongkorn University, Bangkok, 10330 Thailand

**Keywords:** Antivirals, Chikungunya virus, Dual color fluorescent assay, Drug discovery, High-throughput screening, Microbiology, Molecular biology, Medical research

## Abstract

Chikungunya virus (CHIKV), one of the arthropod-borne viruses, has been affecting the global population for more than 70 years since it was first described with more than 620,000 cases in 2024 alone. Despite its long-standing problem, the only treatment available for chikungunya-infected patients is supportive treatment to alleviate pain. Fluorescent molecules have been used in detecting viral infection in the host cells via immunofluorescence assays because of their sensitivity. This study aimed to use this assay to rapidly screen efficacy and cytotoxicity of several compounds in a high-throughput manner. The optimized conditions were to seed Vero cells at 10,000 cells/well, and infect them with CHIKV ECSA at MOI of 0.1. These conditions resulted in a good discrimination power between infected wells and uninfected wells and minimized the cytopathic effect on host cells. Validation using two compounds with known activity against CHIKV, cycloheximide (CHX), and acyclovir (ACY), showed that the assay could properly identify active compounds and inactive compounds correctly. There was also no significant difference between the results of 3 independent rounds of compound screening, thus showing the reproducibility of the assay. Traditional primary screening were performed in parallel with the dual-color fluorescent assay for 60 unknown compounds to evaluate inhibition performance of inhibition and approximate cytotoxicity assessment. The results showed excellent performance from the analysis of the ROC curves and general agreement between two approaches from the Bland-Altman plots. Overall, the developed assay required less labor while being able to screen more compounds than the traditional assay in one round of experiment. The assay is currently being tested to screen libraries of compounds and so far, has been able to identify 22 hits for further characterization.

## Introduction

Chikungunya virus (CHIKV) is an arthropod-borne virus (arbovirus) first identified in Tanzania in 1952 and described in 1955^1,2^. CHIKV has caused a massive outbreak between 2004 and 2007, with more than 1.5 million reported cases in 60 countries in the Indian Ocean region^3^. Subsequent outbreaks have also been reported in Europe^4^ and the United States^5^, establishing CHIKV as a major global public health concern. The predominant circulating strains responsible for these outbreaks belong to the East/Central/South African (ECSA) and Indian Ocean Lineage (IOL) clades^6,7^, with a geographical distribution primarily in tropical regions, aligning with the habitat of the *Aedes albopictus* mosquito^8^.

Chikungunya infection is characterized by arthralgia and fever, with a 44% chance that symptomatic patients become chronic and a 0.3% chance of mortality^9^. During acute infection, viremia was common during days 1 to 7 of illness onset and gradually declined after day 5, with no viral CHIKV viral RNA observed in serum samples after day 14^10^. In addition, viral persistence and inefficient immune responses can contribute to chronic disease. CHIKV RNA could persist in musculoskeletal tissues for at least 16 weeks after onset, contributing to chronic arthritis and tenosynovitis^11^. Although the FDA has approved the first preventive CHIKV vaccines^12^, there is still no specific treatment available for patients. Symptomatic care to alleviate pain is recommended, such as a combination of level 1–2 analgesics and NSAIDs, cryotherapy, and joint immobilization with adequate hydration. Corticosteroids are not recommended during the acute phase, but have an indication for the management of post-acute and chronic infections in combination with other analgesics when NSAID treatment becomes ineffective^13^. Therefore, CHIKV antivirals can be indicated for the treatment of acute infections and to prevent progression to chronic disease.

The current workflow to identify new antiviral drugs typically involves screening candidate compounds for efficacy and cytotoxicity using a combination of virtual screening and cell-based approaches. These potential hits are then characterized (e.g., pharmacokinetics, metabolic pathways) and further evaluated in animal models and clinical trials. Recently, we explored the integration of an immunofluorescence assay (IFA) into the primary screening process to quantify the efficacy of the compound, using a newly developed image analysis algorithm^14^, as this method offers a simplified protocol and automated quantification.

Cell-based immunofluorescence assays (IFA) have been used for antiviral screening against viruses such as yellow fever virus (YFV), dengue virus (DENV), zika virus (ZIKV), and SARS-CoV-2^15–17^. Although a study applied IFA to detect antivirals against CHIKV, YFV, and West Nile virus (WNV)^18^, it did not provide a complete validation of the test conditions. Additionally, the previous study did not evaluate the cytotoxicity of the screened compounds, a critical parameter that can be evaluated primarily by simultaneously staining host cell nuclei to quantify the number of total cells remaining. In this study, our objective was to develop and validate a dual-color fluorescent assay that enables simultaneous evaluation of antiviral efficacy and cytotoxicity within a single workflow. This integrated approach is expected to streamline the screening of high-throughput compounds and accelerate the identification of promising candidates for further development, ultimately contributing to the discovery of effective treatments for CHIKV infection.

## Results

### Development and optimization of the dual-color fluorescent assay

#### Host cell density optimization

**Fig. 1:**
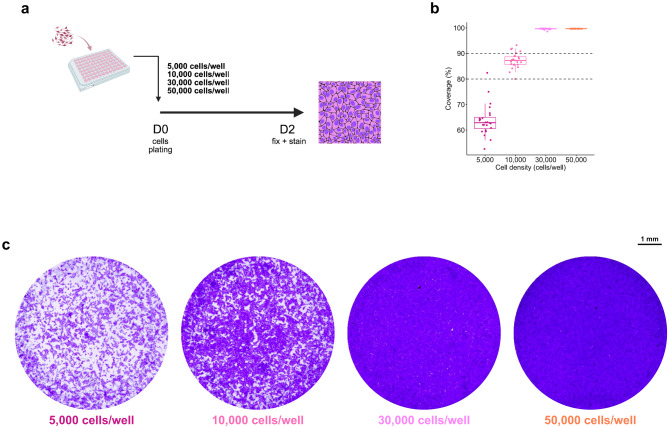
Host cell density optimization. (**a**) Schematic diagram of Vero cells seeded at 5,000, 10,000, 30,000, and 50,000 cells per well and cultured for 48 h. Cells were fixed and stained with 1% crystal violet in formaldehyde. (**b**) Coverage area or cell density were analyzed using Fiji software. Each dot represents the coverage area of each well, and dashed lines represent the target range for density optimization. (*n* = 24). (**c**) Example of wells seeded with Vero cells at various density as specified. Pictures of the well were captured with ImmunoSpot^®^ Analyzers using BioSpot™ software after staining.

Vero cells were selected as the host cell line for CHIKV infection due to their deficiency in interferon production^19^, allowing efficient viral replication. To optimize the seeding density, cells were seeded at 5,000, 10,000, 30,000, and 50,000 cells per well and cultured for 48 h (*n* = 24 per group). Surface coverage was assessed by crystal violet staining and image analysis using Fiji software. The mean confluency ± SD for each density was 63.75 ± 6.08%, 87.28 ± 3.00%, 99.62 ± 0.25%, and 99.69 ± 0.03%, respectively (Fig. 1). A seeding density of 10,000 cells/well, yielding ~ 87% confluency, was selected for further experiments to ensure uniform infection while avoiding overconfluency, which can compromise cell function and viral infectivity.

#### Multiplicity of viral infection (MOI) optimization

**Fig. 2:**
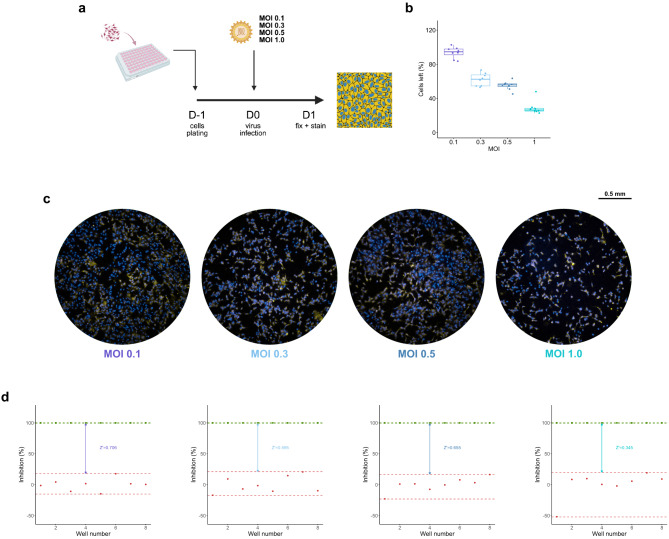
Multiplicity of infection (MOI) of CHIKV ECSA optimization in Vero. (**a**) Schematic diagram of Vero (10,000 cells/well) infected with CHIKV at MOIs of 0.1, 0.3, 0.5, and 1.0 for 24 hours (n = 8 per group). (**b**) Percentage of cells left in wells infected with CHIKV ECSA. Each dot represents the percentage of the number of host cells left in each well. (**c**) Example of wells infected with various concentrations of viruses as specified. The pictures were taken with ImmunoSpot^®^ analyzers using Fluoro-X™ FluoroSpot software after DAPI and immunofluorescent staining of viruses. (**d**) Corresponding value of the Z’ factor with each virus concentration. The green dots represent inhibition of uninfected wells, the red dots represent inhibition of infected wells, the green dashed lines represent variation of inhibition in uninfected wells, the red dashed lines represent variation of inhibition in infected wells, and the arrows represent the power of discrimination between uninfected and infected wells. (*n* = 8).

To determine the optimal MOI, Vero (10,000 cells / well) was infected with CHIKV at MOIs of 0.1, 0.3, 0.5, and 1.0 for 24 h (*n* = 8 per group). Wells with uninfected Vero cells were included as the uninfected control. Infected and total cell counts were quantified by polyclonal antibody together with DAPI staining and image analysis using a newly developed algorithm^14^. The percentage of total cells left and CHIKV inhibition were analyzed per MOI (Fig. 2). The mean percentage of cells left ± SD was 93.68 ± 6.63%, 62.10 ± 7.55%, 55.11 ± 5.31%, and 28.92 ± 3.99% for increasing MOI. The Z ′ factor was calculated to evaluate the discrimination power between infected and uninfected wells^20^, yielding Z′ values of 0.706, 0.595, 0.655, and 0.345, for increasing MOIs. The Z′ value > 0.5 indicated low variability, which resulted in an excellent assay that could separate between infected and uninfected conditions. MOI 0.1 was chosen for subsequent testing due to the minimal cytopathic effect and the excellent discrimination power.

### Validation of conditions

#### Evaluation using reference compounds

**Fig. 3:**
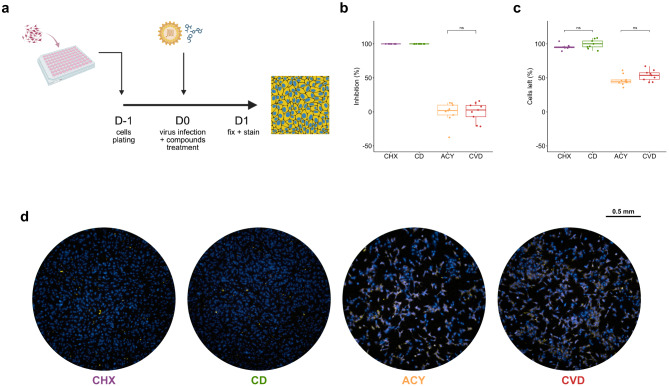
Validation of the dual-color fluorescent assay using compounds with known activity against CHIKV. (**a**) Schematic diagram of Vero (10,000 cells/well) infected with CHIKV (MOI of 0.1). (**b**) Inhibition of each well treated with the specified compounds; CHX/purple = cycloheximide, CD/green = uninfected control (**C**ell + **D**MSO). ACY/yellow = acyclovir, CVD/red = infected control (**C**ell + **V**irus + **D**MSO). (**c**) Percentage of cells left in each well treated by the specified compounds. “ns” represents no statistical significance. (*n* = 9). CHX and CD inhibition showed zero variation, thus ineligible for applying any statistical evaluation. (**d**) Example of the wells treated with compounds as specified. The pictures were taken with ImmunoSpot^®^ Analyzers using Fluoro-X™ FluoroSpot software after DAPI staining and immunofluorescent staining of viruses.

To validate the assay’s ability to discriminate active from inactive compounds, cycloheximide (CHX), a known inhibitor of eukaryotic translation^21^, was used as a positive control, while acyclovir (ACY), an HSV-specific antiviral^22^, served as a negative control. Infected (CVD: **C**ells + **V**irus + **D**MSO) and non-infected (CD: **C**ells + **D**MSO) controls were included to account for baseline responses for inhibition and percentage of total cells left, respectively (*n* = 9 per group). Under optimized conditions, CHX achieved 100.00 ± 0.00% inhibition with 95.51 ± 3.83% cells left, while ACY resulted in − 0.84 ± 15.67% inhibition and 46.47 ± 7.82% cells left (Fig. 3). CHX treated wells did not show significant differences from uninfected controls (*p* = 0.100 for the percentage of cells left), while ACY treated wells did not differ significantly from the infected controls (*p* = 0.906 for inhibition; *p* = 0.079 for percentage of cells left). Statistical testing was not applied to CHX inhibition due to zero variance. These results demonstrated the power of discrimination between active and inactive compounds.

#### Reproducibility of the assay

**Fig. 4:**
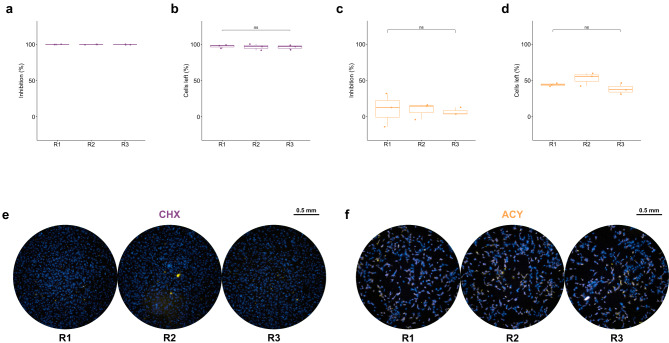
Reproducibility of the assay evaluated by 3 rounds (R1–3) of CHX and ACY screening. Each dot represents data from each well, CHX/purple = cycloheximide and ACY/yellow = acyclovir. (**a**) Inhibition of CHX-treated wells between each round. (**b**) Percentage of cells left in CHX-treated wells between each round. (**c**) Inhibition of ACY-treated wells between each round. (**d**) Percentage from cells left of ACY-treated wells between each round. “ns” represents no significance. (*n* = 9). No statistical tests were performed for CHX inhibition as there was no variation. (**e**) Example of the wells treated with CHX between each round. (**f**) Example of wells treated with ACY between each round. The pictures were taken with ImmunoSpot^®^ Analyzers using Fluoro-X™ FluoroSpot software after DAPI staining and immunofluorescent staining of viruses.

Reproducibility was evaluated in three independent assay rounds using CHX and ACY (*n* = 3 per group per round) (Fig. 4). CHX consistently showed 100.00 ± 0.00% inhibition, with 97.51 ± 2.47%, 96.58 ± 4.30%, and 90.68 ± 3.04% of the cells left. ACY exhibited low inhibition values—10.26 ± 23.12%, 8.96 ± 11.15%, and 6.84 ± 5.33%—with corresponding 39.25 ± 9.32%, 46.99 ± 0.93%, and 40.70 ± 8.58% of cells left. No significant variation was observed in three rounds (ANOVA: CHX, *p* = 0.091 for the percentage of cells left; ACY, *p* = 0.962 for the inhibition and 0.332 for percentage of cells left). Statistical testing was not applied to CHX inhibition due to zero variance. These results confirmed the reproducibility of the assay.

#### Performance comparison with standard assay

**Fig. 5:**
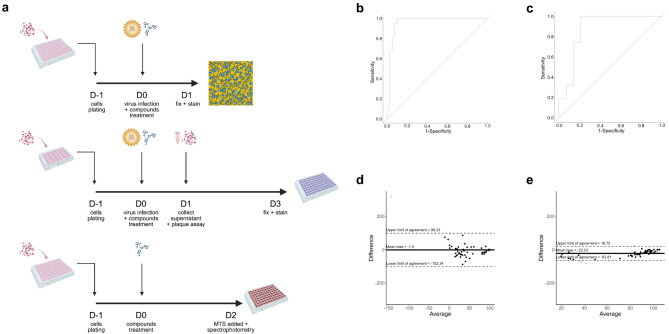
Performance comparison between dual color fluorescence assay and 2 standard methods: plaque reduction assay for inhibition assessment and MTS viability assay for viability assessment. (**a**) Schematic diagrams of dual color fluorescent assay, plaque reduction assay, and MTS viability assay, from top to bottom. (**b**) ROC curve of inhibition assessed by a dual color fluorescent assay compared to the results of the plaque inhibition assay. (**c**) ROC curve of the percentage of cells left assessed by a dual color fluorescent assay compared to the results from the MTS viability assay. (**d**) Bland-Altman plots of differences in the calculated inhibition compared to plaque reduction assay. (**e**) Bland-Altman plots of the differences in the calculated percentage of cells left compared to the MTS viability assay. (For Bland-Altman plots; black dots represent the difference between the readouts of both approaches that are still within the limit of agreement, gray dots represent the difference between readouts of both approaches that fall outside the limit of agreement, thick line represents the mean bias of the dual color fluorescent assay, and dashed lines represent the range of the limit of agreement (± 1.96SD)).

To benchmark the high-throughput dual-color fluorescent assay, 60 compounds (in triplicate) were screened in parallel using standard methods: plaque assay for CHIKV inhibition and MTS assay for cell viability (Fig. 5a), to confirm whether the percentage of cells left can be used to approximate assess cytotoxicity of the compounds or not (Table S2-31). Given the different detection mechanisms, results were compared using receiver operating characteristic (ROC) curve^23^ and Bland-Altman analyzes^24^ to evaluate classification performance and agreement between methods.

##### ROC curve analysis

For inhibition, the area under the curve (AUC), calculated using the DeLong method, was 0.962 (95% CI: 0.911–1.012), indicating excellent performance. The optimal cutoff point for inhibition was 72.54%, based on the Youden index. However, an 80% cutoff was adopted for the actual screening to increase the stringency (Fig. 5b).

For viability, the AUC was 0.876 (95% CI 0.732–1.019), also indicated excellent performance and showed that the number of cells remaining can be used to approximate the viability. Although the optimal cut-off point was 44.55%, an 80% threshold was again used for consistency and conservative screening (Fig. 5c).

##### Bland-Altman plots

For inhibition, the mean bias was -1.50%, indicating slight underestimation by the dual color fluorescence assay. Only one point of the 60 screened compounds fell outside the agreement limits (-102.24–99.23%), indicating overall agreement between the two approaches (Fig. 5d).

For viability, the mean bias was -22.53%, again showing underestimation by the dual color assay. Four data points out of the 60 compounds screened fell outside the agreement range (-63.81–18.75%) (Fig. 5e). Overall, these results demonstrate that the assay performs with acceptable agreement with the standard methods.

### Application of the dual-color fluorescent assay

**Fig. 6:**
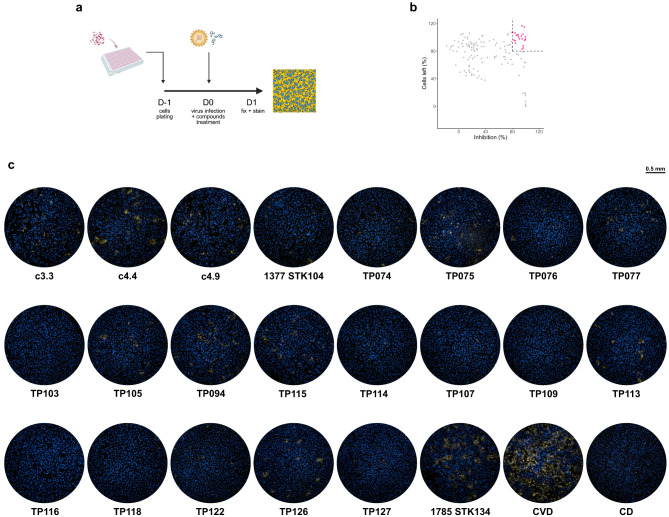
Current results from compounds library screening. (**a**) Schematic diagram. (**b**) Inhibition and cell left results from all compounds that were screened by the dual-color fluorescent assay. Gray dots represent mean inhibition and percentage of cells left of compounds that did not pass the 80% cutoff, pink dots represent mean inhibition and percentage of cells left of compounds that passed the 80% cutoff, and black dashed lines represent the cutoff point at 80%. (**c**) Example of wells treated with hits compared to infected control (CVD) and uninfected control (CD). The pictures were taken with ImmunoSpot^®^ Analyzers using Fluoro-X™ FluoroSpot software after DAPI staining and immunofluorescent staining of viruses.

The optimized dual-color fluorescence assay is currently being tested for routine high-throughput screening of anti-CHIKV compounds. In this report, 150 compounds have been tested, yielding 22 hits that meet the predefined criteria of > 80% inhibition and > 80% cells left (Fig. 6, Table S2-37). These compounds are currently being investigated further for potential development as anti-CHIKV therapeutics.

## Discussion

This study has successfully developed, optimized, and validated a dual color fluorescence assay for high-throughput screening of anti-CHIKV compounds. Although similar approaches have been used in prior studies^18^, few have rigorously evaluated the reliability of the test conditions. Our results confirm that lower multiplicities of infection (MOIs), such as 0.1, 0.3, and 0.5, provide comparable discrimination between infected and uninfected cells. However, the use of a lower MOI (0.1) significantly reduces CHIKV-induced cytopathic effects, thus improving the consistency and reliability.

The Vero cell line was selected for this platform because of its inherent capacity to support rapid viral replication, attributed to its deficiency in interferon production, as previously discussed. This characteristic makes Vero cells suitable for high-throughput platforms that require a rapid turnaround in drug discovery. However, Vero cells are not the natural host of the chikungunya virus. To enhance the translational relevance of this platform, the inclusion of primary cells or adaptation to human derived cell lines, such as Huh-7 hepatoma cells, human dermal fibroblasts, or neural progenitor cells, could also enhance the translational relevance of the assay, allowing a more accurate assessment of antiviral efficacy and cytotoxicity in a physiologically relevant context. However, the infectivity rate of these cells might be less robust than that of Vero cells due to their intact cellular immunity, thus affecting the Z’ score, or the power of discriminating potent and impotent compounds.

Cell culture experiments are inherently variable, as demonstrated by the differing percentage of cells left observed under similar conditions. For example, infected wells at MOI 0.1 resulted in 93.68 ± 6.63% cells left in the well during optimization, while the infected control during assay validation using reference compounds resulted in only 53.46 ± 7.98% cells left. Such discrepancies are expected due to experimental variability, highlighting the importance of minimizing virus-induced cytotoxicity by choosing an MOI that consistently maintains a sufficient number of host cells left. We determined that if the percentage of host cells left in the infected control remains above or equal to 50%, the assay readouts are still usable (as the MOI of 0.5 still resulted in excellent discrimination power with 55.11 ± 5.31% of the cells left). Therefore, each experiment should routinely be assessed whether the percentage of host cells falls below this threshold; if it does, the virus stock should be re-titrated, and the experiment repeated.

As shown in the experiment that more than 90% of cells are still left after being treated with cycloheximide, a known toxic compound, there are multiple factors that can play into the number of cells that remain attached to the well surface. That is why even though the ROC analysis and the Bland-Altman plot showed that the percentage of host cells left can be used to approximate the compound’s cytotoxicity, or in other words, the viability of the host cells, the cytotoxicity should also be reconfirmed through standard methods. To isolate compound-specific cytotoxicity from virus-induced effects, we tried to select only compounds that had more than 80% virus inhibition to compare cytotoxicity assessment between using the percentage of cells left and the MTS viability assay. Using this method improved viability assessment performance, as evidenced by an increase in the AUC of the ROC curve to 0.933 (95% CI: 0.799–1.068), with only one outlier in the Bland-Altman analysis.

Another important internal control is the Z’ factor, a widely accepted parameter for evaluating the robustness of high-throughput assays^20^. For each experiment, a Z’ factor above 0 is acceptable for binary screening, as only triplicates of control could result in a higher variability. However, aggregated data from multiple experiments should yield a Z’ factor above 0.5 for the overall robustness of the assay. In our study, compiling results from 41 control replicates produced a Z’ factor of 0.634, confirming strong overall assay performance in distinguishing between infected and uninfected conditions.

Compared to traditional methods, this dual-color fluorescence assay offers several advantages. Although plaque reduction assays are commonly used due to their low cost, they are indirect, labor intensive, and subject to variability in plaque shape and size. IFA, on the other hand, provide greater sensitivity by detecting individual viral particles. Our dual color assay builds on this by adding a nuclear stain (DAPI) to simultaneously assess the number of remaining cells. Since Vero cells adhere to the substrate, dead cells are washed away during processing, which means that DAPI-positive cells should represent mostly viable cells. 

Furthermore, the test improves throughput and efficiency. A technician who handled six plates for three days could screen 180 compounds and obtain both efficacy and cytotoxicity data using our method. On the contrary, a traditional plaque assay would take at least five days to produce efficacy data for only 108 compounds and would require additional plates and time for parallel MTS viability testing. Ultimately, the traditional method would require 24 plates (18 for efficacy, 6 for viability) compared to just 3 plates using our assay, demonstrating substantial reductions in workload and time.

Despite its advantages, the assay has limitations. One challenge is that virus particles are too small to be counted directly, unlike host nuclei stained with DAPI. Our algorithm bypasses this by detecting infected cells through viral signals surrounding the nucleus instead^14^, providing a practical proxy for the level of infection. Furthermore, inhibition efficiency is gauged by counting E1-labeled infected cells still adhere to the well, relative to the average count of infected cells in the untreated control. Since the E1 protein serves as an indicator of infection, compounds that stop viral mRNA at an early stage will exhibit a high inhibition. The cycloheximide control demonstrated this effect, though prolonged use of cycloheximide can be toxic to host cells.

As this assay was designed for primary screening, all compounds were tested at a fixed concentration and applied both during and after infection. This simulates therapeutic use by assessing compounds for already-infected patients. However, more assays are needed to fully characterize hits, determining IC50, CC50, time-of-addition, and mechanism of action. Currently, these follow-up assays are based on traditional methods such as plaque and MTS assays. A future goal is to extend the dual-color assay platform to include these post-screening evaluations using the same fluorescent markers and image analysis tools.

In summary, this study establishes a comprehensive framework for the development of high-throughput antiviral screening assays. It includes defining optimal cell and virus densities, validating assay conditions with known compounds, evaluating reproducibility, and benchmarking performance against traditional methods. This workflow can be adapted to develop similar assays for other viruses, efforts are already underway for Dengue and Zika, or inspire the creation of novel high-throughput screening strategies.

## Materials and methods

### Cells and viruses

C6/36 (ATCC^®^ CRL-1660) and Vero (ATCC^®^ CCL-81) cell lines were cultured in growth medium (Gibco^®^, Langley, OK, USA) supplemented with fetal bovine serum (FBS) at 10% for C6/36 and 5% for Vero cells, along with 100 I.U./mL penicillin and 100 µg/mL streptomycin (Bio Basic Canada^®^, Ontario, Canada), and 10 mM HEPES (Sigma Aldrich^®^, St. Louis, MO, USA). Vero cells were maintained at 37 °C with 5% CO_2_, whereas C6/36 cells were maintained at 28 °C. The East/Central/South African (ECSA) chikungunya virus (CHIKV) was propagated in C6/36 cells using a maintenance medium supplemented with 1% FBS, antibiotics and HEPES. All cells were routinely passaged 2 times a week with 0.05% trypsin-EDTA for 2–3 min, 90% of the old cells were discarded, then the leftover cells were replenished with fresh media.

### Plaque Titration assay

Vero cells (5 × 10^4^ cells/well) were seeded in 24 well plates and incubated for 24 h at 37 °C, 5% CO_2_. Ten-fold serial dilutions of propagated CHIKV ECSA (up to 10^− 10^) were prepared in maintenance medium and used to infect monolayers in duplicate. After an 1-hour infection period, the viral inoculum was removed and cells were washed with phosphate buffered saline (PBS), then overlaid with medium containing 1% FBS, antibiotics, 10 mM HEPES, and 1.2% gum tragacanth (Sigma Aldrich^®^). The plates were incubated for 24 h, after which the cells were fixed and stained with a solution of 25% formaldehyde (Carlo Erra^®^, Milan, Italy), 5% isopropanol (Merck^®^, Darmstadt, Germany), and 1% crystal violet (Merck^®^). Plaques were manually counted to determine plaque-forming units per milliliter (p.f.u./mL).

### Dual-color fluorescent high-throughput screening assay

This assay was adapted from the cell-based flavivirus infection (CFI) protocol^25^. Vero cells (1 × 10^4^ cells/well) were seeded in 96-well plates and incubated for 24 h. On day two, cells were infected with CHIKV ECSA at a multiplicity of infection (MOI) of 0.1 for 1 h. After virus removal and washing with PBS, a maintenance medium was added for an additional 24 h incubation. Compounds diluted in 1% DMSO at a final concentration of 10 µM were applied in triplicate both during and after infection. Infected cells treated with 1% DMSO served as the infected control (0% inhibition) control; uninfected cells treated with 1% DMSO served as the uninfected control (100% cell left). On day three, cells were fixed with 10% neutral buffered formalin (NBF), permeabilized with 0.1% Triton X-100 (Sigma Aldrich^®^) and blocked with 2% bovine serum albumin (BSA) (Capricorn Scientific^®^, Ebsdorfergrund, Germany) for 30 min each. The CHIKV E1 antigen was detected using a polyclonal anti-E1 primary antibody (Thermo Fisher Scientific^®^, #PA5-117442, 1:500) followed by a secondary antibody conjugated with Alexa Fluor TM 568 goat anti-rabbit IgG secondary antibody (Thermo Fisher Scientific^®^, #A-11036, 1:500) and nuclear staining with DAPI (1:10,000). All of the staining reagents were diluted in 0.1% BSA and incubated for 1 h, with PBS washes between each step, ending with a final wash in PBS containing 0.1% Tween 20. Plates were air-dried and stored at 4°C, wrapped in foil, until imaging.

### Post-imaging analysis

Images were acquired using ImmunoSpot^®^ Analyzers (Cellular Technology Limited, Ohio, USA) and Fluoro-X™ FluoroSpot software at 4.48× magnification. A custom automated image processing algorithm^14^ was used to quantify infected and total cells. Inhibition and percentage of cells left were calculated as:

$$\:\text{i}\text{n}\text{h}\text{i}\text{b}\text{i}\text{t}\text{i}\text{o}\text{n}=\left(1-\frac{{\text{I}}_{\text{w}\text{e}\text{l}\text{l}}}{{{\upmu\:}\text{I}}_{\text{C}\text{V}\text{D}}}\right)\times\:100$$

$$\:\text{c}\text{e}\text{l}\text{l}\text{s}\:\text{l}\text{e}\text{f}\text{t}=\left(\frac{{\text{N}}_{\text{w}\text{e}\text{l}\text{l}}}{{{\upmu\:}\text{N}}_{\text{C}\text{D}}}\right)\times\:100$$

where I_well_ represents the number of infected cells in that well, µI_CVD_ represents the average number of infected cells in the infected control (**C**ells + **V**iruses + **D**MSO), N_well_ represents the number of total cells in that well, µN_CD_ represents the average number of total cells in the uninfected control (**C**ells + **D**MSO).

### Development and optimization of the dual-color fluorescent assay

#### Density of the host cells

Vero cells were seeded in 96-well plates at densities of 5 × 10^3^, 1 × 10^4^, 3 × 10^4^, and 5 × 10^4^ cells/well (*n* = 24 per condition) and incubated for 24 h. The medium was then replaced with a maintenance medium and incubated overnight. On day three, cells were fixed and stained with formaldehyde, isopropanol, and crystal violet for 1 h, washed, and air-dried. The images were captured by ImmunoSpot^®^ Analyzers using BioSpot™ software before being turned into 8-bit pictures for manual thresholding and analysing percentage of coverage area by Fiji software.

#### Multiplicity of viral infection optimization

Vero cells in growth media were seeded at 1 × 10^4^ cells per well in a 96-well plate and incubated for 24 hours to let the cells attach to the surface. CHIKV ECSA in maintenance media was prepared at MOI of 0.1, 0.3, 0.5, and 1.0 to infect the cells on the second day for 1 hour (n = 8 per condition). The viruses were aspirated out and the cells were then washed with PBS and kept in maintenance media for 24 hours. Uninfected Vero cells treated with 1% DMSO were used as an uninfected control for Z’ factor calculation. On the third day, the plate was processed, before the images were captured and analyzed as described. Afterward, calculated cell left was used to assess cytopathic effect of the virus, and calculated inhibition was used to further calculate the Z’ factor to assess the discrimination power between infected and uninfected wells of each MOI using the formula:

$$\:{\text{Z}}^{{\prime\:}}\text{f}\text{a}\text{c}\text{t}\text{o}\text{r}=1-\frac{{3{\upsigma\:}\text{I}}_{\text{C}\text{V}\text{D}}+{3{\upsigma\:}\text{I}}_{\text{C}\text{D}}}{\left|{{\upmu\:}\text{I}}_{\text{C}\text{V}\text{D}}-{{\upmu\:}\text{I}}_{\text{C}\text{D}}\right|}$$

where σI_CVD_ and σI_CD_ represent the standard deviation of the number of infected cells in the infected wells and uninfected wells respectively, and µI_CVD_ and µI_CD_ represent the average number of infected cells in the infected wells and uninfected wells respectively.

### Validation of assay conditions

#### Evaluation using reference compounds

Vero cells were infected with CHIKV ECSA at MOI 0.1 and treated with cycloheximide (CHX; positive control) and acyclovir (ACY; negative control) at 10 µM (*n* = 9). Infected and uninfected controls were treated with 1% DMSO. On day three, the assay was completed as described. Statistical significance between positive control and non-infected control, and negative control and infected control was assessed using a two-tailed Student’s t test.

#### Reproducibility of the assay

CHX and ACY treatments were repeated in three independent experiments (*n* = 3 per round). The inhibition and percentage of cells left were analyzed by one-way ANOVA to evaluate the reproducibility.

#### Performance comparison with standard assays

##### Plaque reduction assay

Vero cells (5 × 10^4^ cells/well, 24-well plates) were infected with CHIKV ECSA at MOI 0.1 and treated with 60 randomly selected compounds (10 µM in 1% DMSO). The supernatants were collected after 24 h, serially diluted to 10^− 3^, and used to infect fresh Vero cells. After 1 h of incubation, an overlay medium was added. At 48 h after infection, cells were fixed, stained and plaques were manually counted to determine inhibition.

##### MTS viability assay

Vero cells (1 × 10^4^ cells/well, 96-well plates) were treated with the same 60 compounds in 1% DMSO (10 µM) for 48 h. MTS reagent (10%) was added and incubated for 3 h. The absorbance at 490 nm was measured and compared to the DMSO controls to determine viability.

##### ROC curve analysis

Traditional plaque reduction and MTS viability assays served as gold standards. Cutoffs of ≥ 90% inhibition and ≥ 95% viability were used as “case” for the traditional assays. Receiver Operating Characteristic (ROC) curves for dual-color assay performance were generated using easyROC^26^.

##### Bland-altman plots

The differences between dual-color and conventional assays were calculated and mean bias and 95% limits of agreement were determined. Bland-Altman plots were generated by plotting average values on the x-axis and differences on the y-axis, with mean bias and confidence intervals visualized.

## Supplementary Information

Below is the link to the electronic supplementary material.

Supplementary Material 1

## Data Availability

The datasets used and/or analyzed during the current study are available from the corresponding author on reasonable request.
