# Supplementary material for: Development, validation, and application of a dual-color fluorescent assay for high-throughput screening of anti-chikungunya drugs
Source: Sci Rep. 2025 Aug 22;15:30860. doi: 10.1038/s41598-025-16087-1 (PMC12373836; doi:10.1038/s41598-025-16087-1)
Supplement: Supplementary file 1 — Supplementary Material 1 [file 41598_2025_16087_MOESM1_ESM.pdf]

Development, validation, and application of a dual-color fluorescent assay for high-throughput screening of anti-chikungunya drugs

Pattadon Sawetpiyakul[1,2], Duangpron Peypala[1], Pathaphon Wiriwithya[1,3], Gridsada Panomchoeng[1,3], Tanatorn Khotavivattana[4], Warintorn Chavasiri[4], Sittiporn Pattaradilokrat[2] & Siwaporn Boonyasuppayakorn[1]*

[1]Center of Excellence in Applied Medical Virology, Department of Microbiology, Faculty of Medicine, Chulalongkorn University, Bangkok, 10330, Thailand

[2]Department of Biology, Faculty of Science, Chulalongkorn University, Bangkok, 10330, Thailand

[3]Department of Mechanical Engineering, Faculty of Engineer, Chulalongkorn University, Bangkok, 10330, Thailand

[4]Center of Excellence in Natural Product, Department of Chemistry, Faculty of Science, Chulalongkorn University, 10330, Thailand

*Corresponding to Siwaporn.b@chula.ac.th

Supplementary

Supplementary 1 Pictures of experimented plates

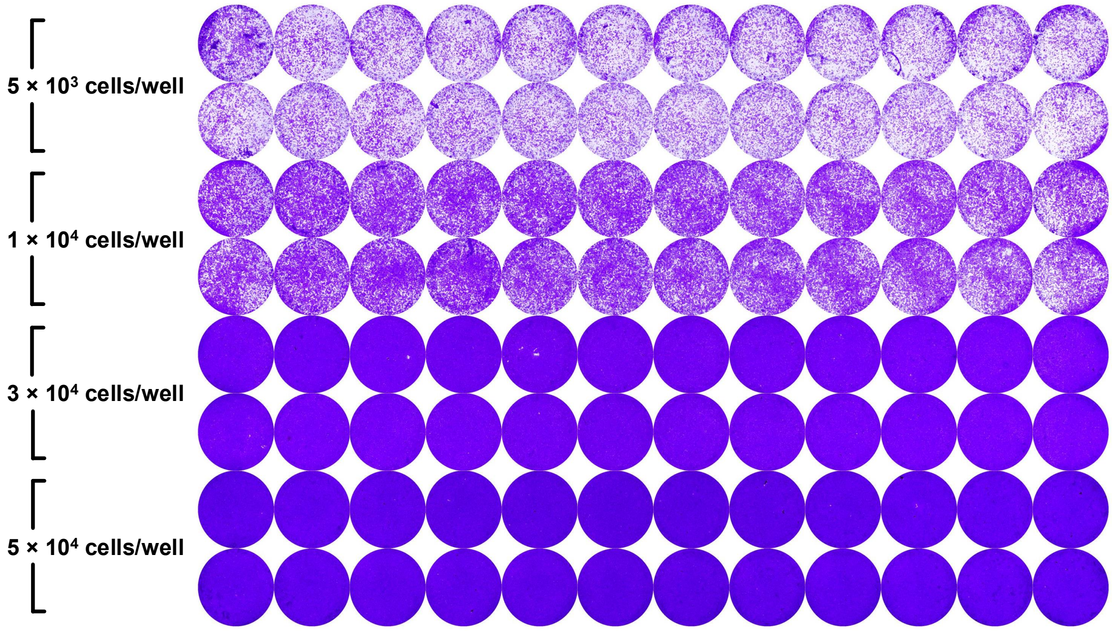

Figure S1-1 Crystal violet-stained Vero cells seeded at various densities for cell optimization. Pictures of the whole well were captured with ImmunoSpot® Analyzers using BioSpot™ software.

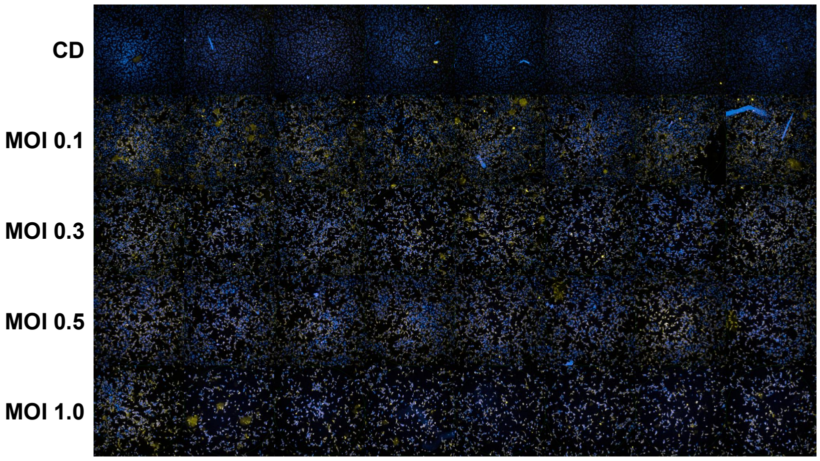

Figure S1-2 CHIKV ECSA-infected Vero cells for MOI optimization. CD=uninfected control (Cell+DMSO). Pictures were taken with ImmunoSpot® Analyzers using Fluoro-X™ FluoroSpot software at 4.48× magnification.

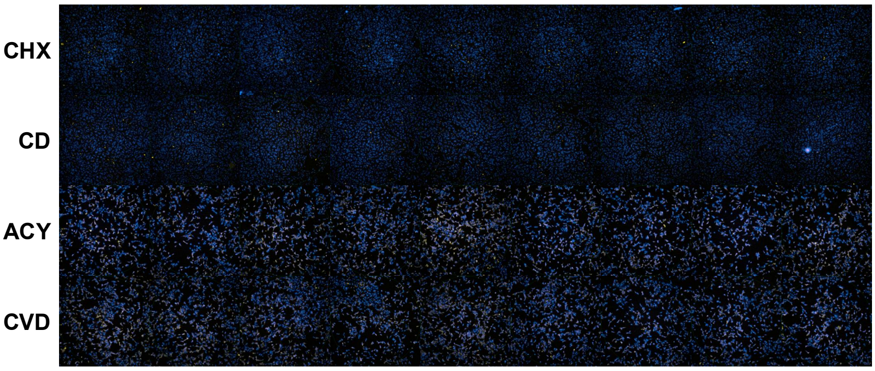

Figure S1-3 Validation using known compounds. CHX=cycloheximide-treated, CD=uninfected control (Cell+DMSO), ACY=acyclovir-treated, CVD=infected control (Cell+Virus+DMSO).

Pictures were taken with ImmunoSpot® Analyzers using Fluoro-X™ FluoroSpot software at 4.48× magnification.

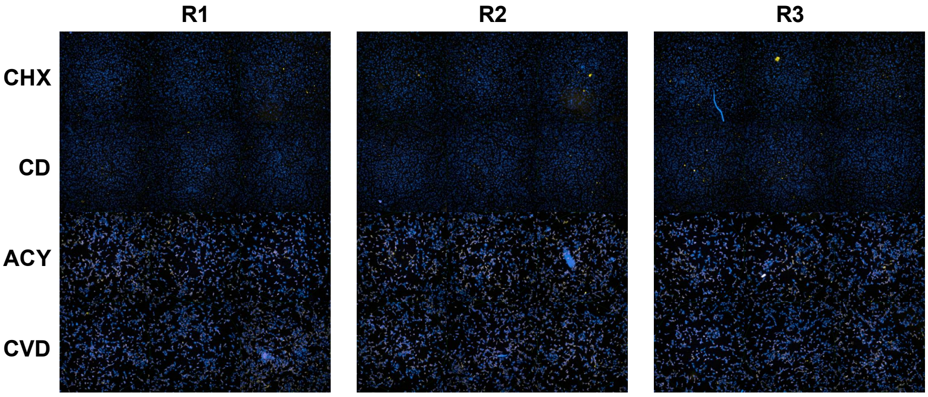

Figure S1-4 Repeatability assessment using known compounds. R=round of screening.

Pictures were taken with ImmunoSpot® Analyzers using Fluoro-X™ FluoroSpot software at 4.48× magnification.

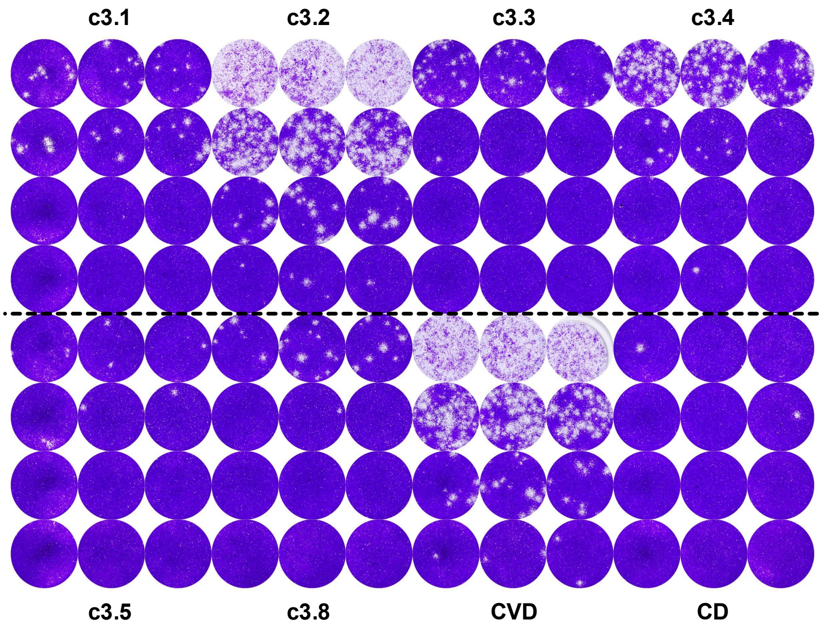

Figure S1-5 Plaque screening of unknown compounds as specified, plate 1.

Pictures of the whole well were captured with ImmunoSpot® Analyzers using BioSpot™ software.

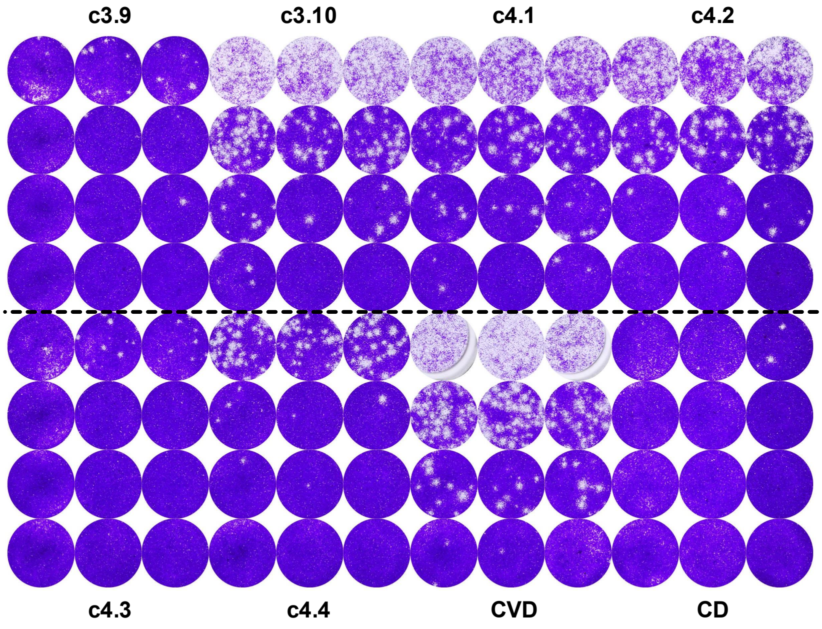

Figure S1-6 Plaque screening of unknown compounds as specified, plate 2.

Pictures of the whole well were captured with ImmunoSpot® Analyzers using BioSpot™ software.

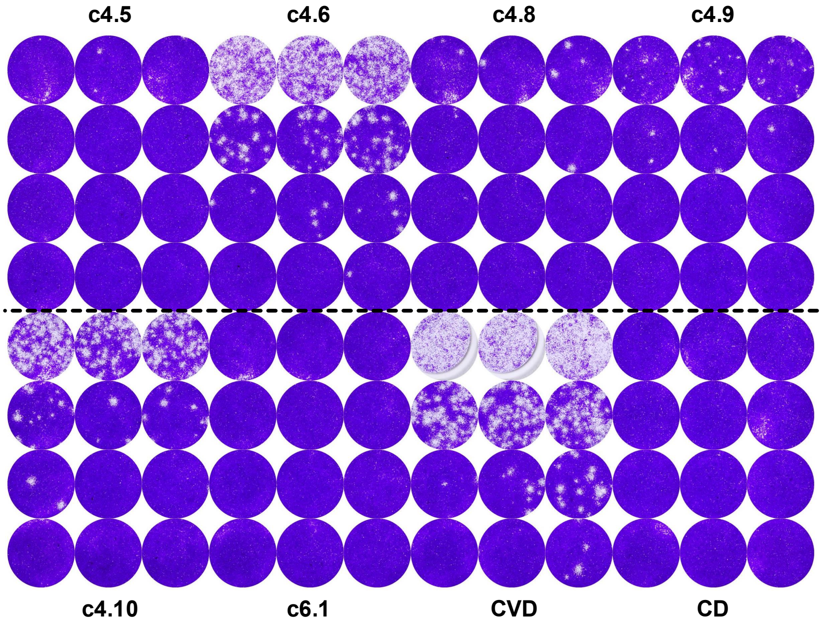

Figure S1-7 Plaque screening of unknown compounds as specified, plate 3.

Pictures of the whole well were captured with ImmunoSpot® Analyzers using BioSpot™ software.

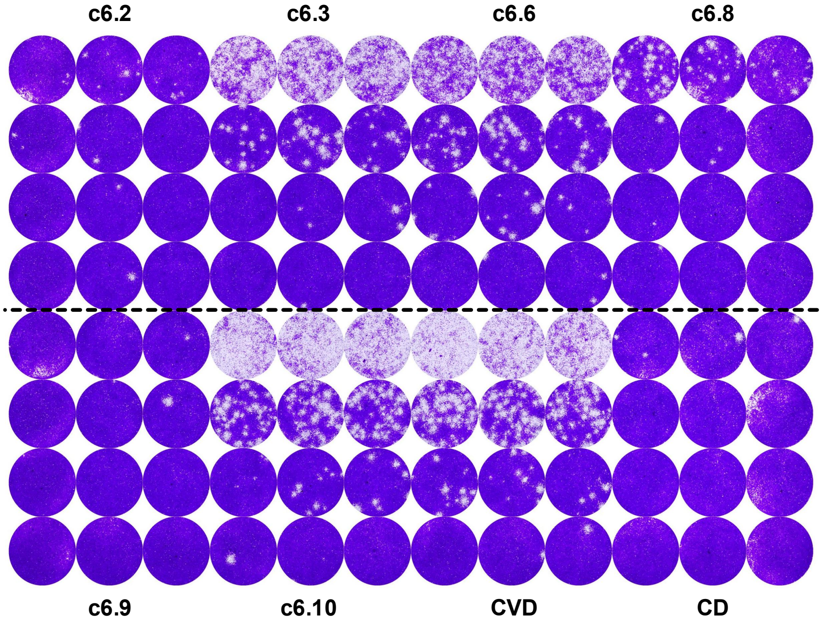

Figure S1-8 Plaque screening of unknown compounds as specified, plate 4.

Pictures of the whole well were captured with ImmunoSpot® Analyzers using BioSpot™ software.

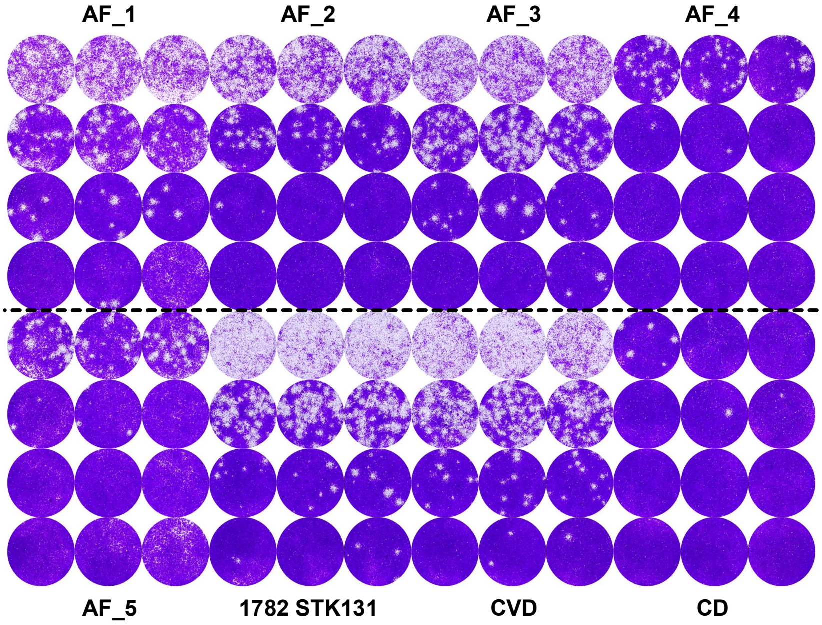

Figure S1-9 Plaque screening of unknown compounds as specified, plate 5.

Pictures of the whole well were captured with ImmunoSpot® Analyzers using BioSpot™ software.

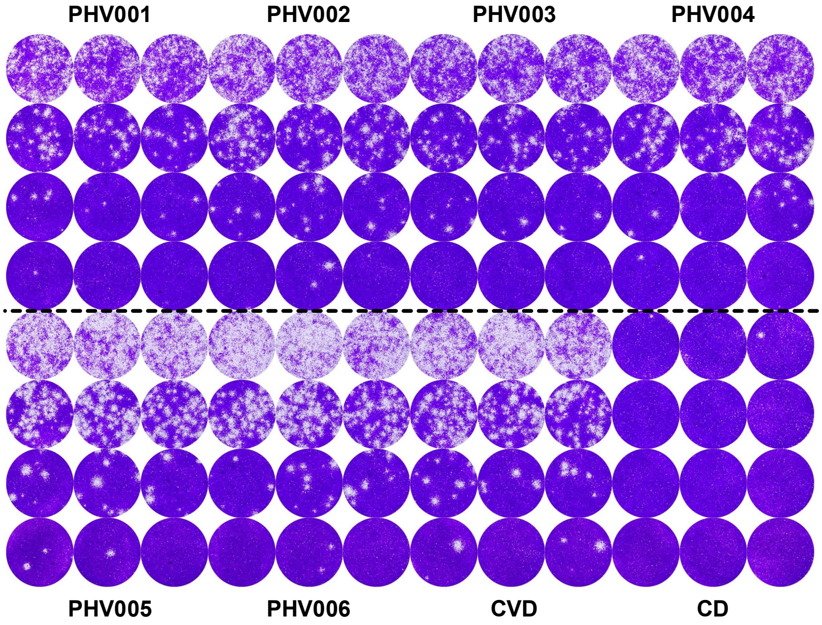

Figure S1-10 Plaque screening of unknown compounds as specified, plate 6.

Pictures of the whole well were captured with ImmunoSpot® Analyzers using BioSpot™ software.

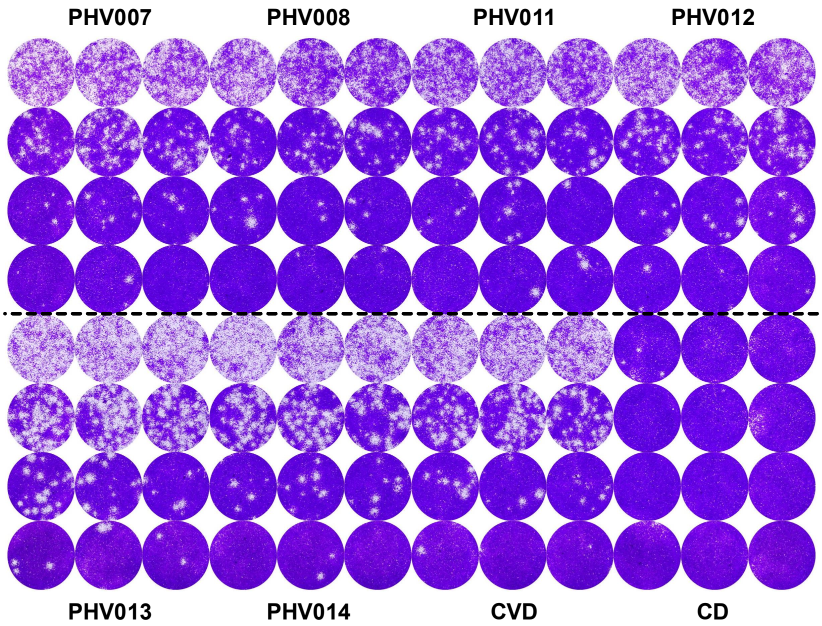

Figure S1-11 Plaque screening of unknown compounds as specified, plate 7.

Pictures of the whole well were captured with ImmunoSpot® Analyzers using BioSpot™ software.

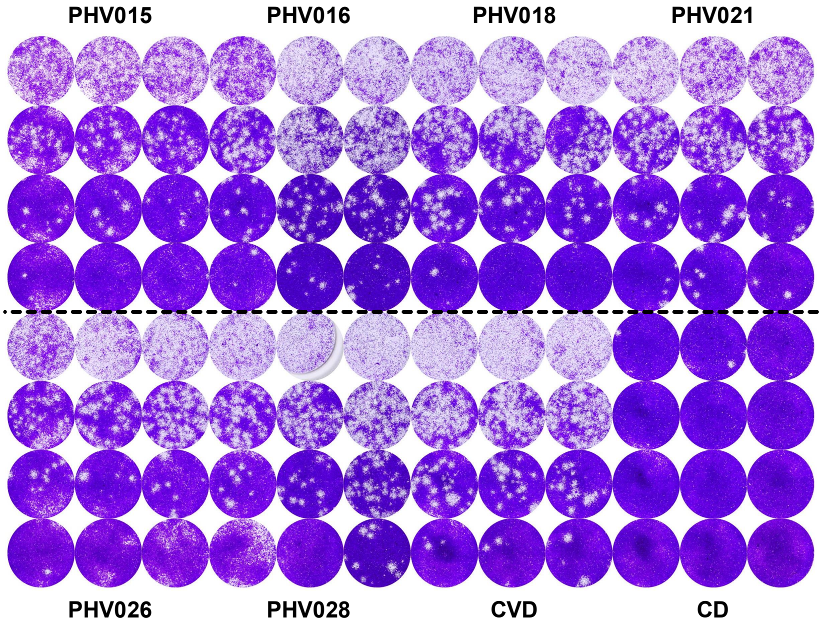

Figure S1-12 Plaque screening of unknown compounds as specified, plate 8.

Pictures of the whole well were captured with ImmunoSpot® Analyzers using BioSpot™ software.

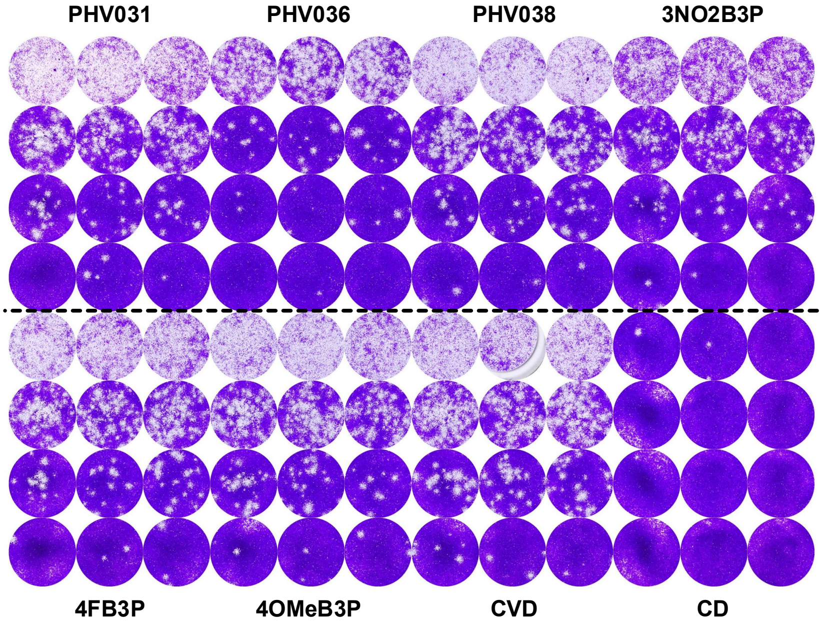

Figure S1-13 Plaque screening of unknown compounds as specified, plate 9.

Pictures of the whole well were captured with ImmunoSpot® Analyzers using BioSpot™ software.

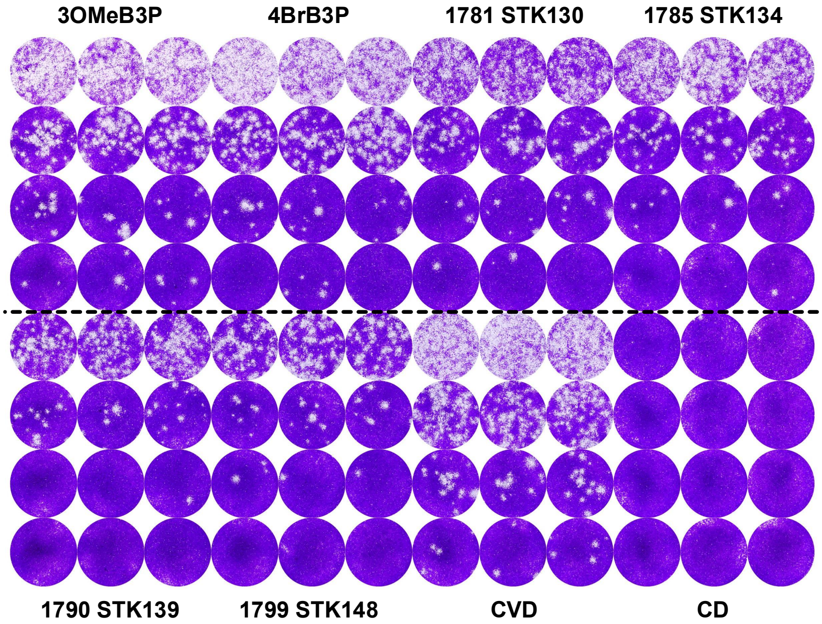

Figure S1-14 Plaque screening of unknown compounds as specified, plate 10.

Pictures of the whole well were captured with ImmunoSpot® Analyzers using BioSpot™ software.

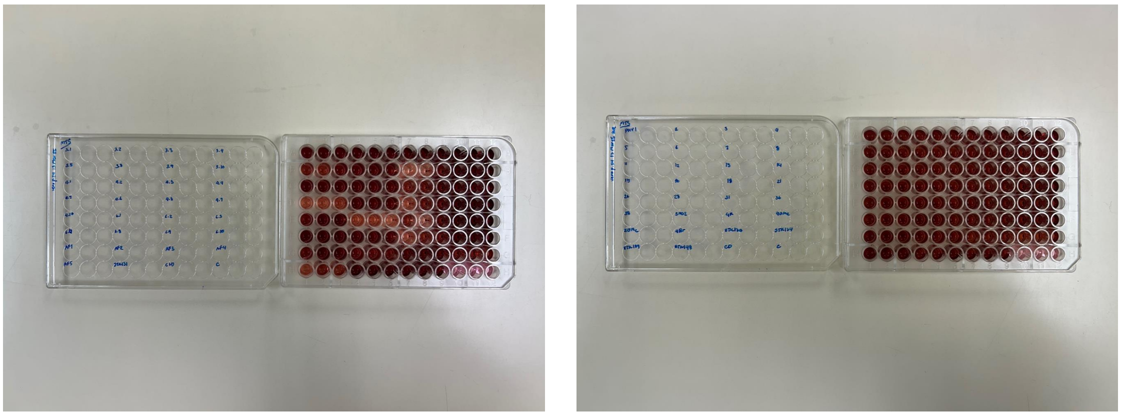

Figure S1-15 MTS viability screening of unknown compounds as specified on the plate cover.

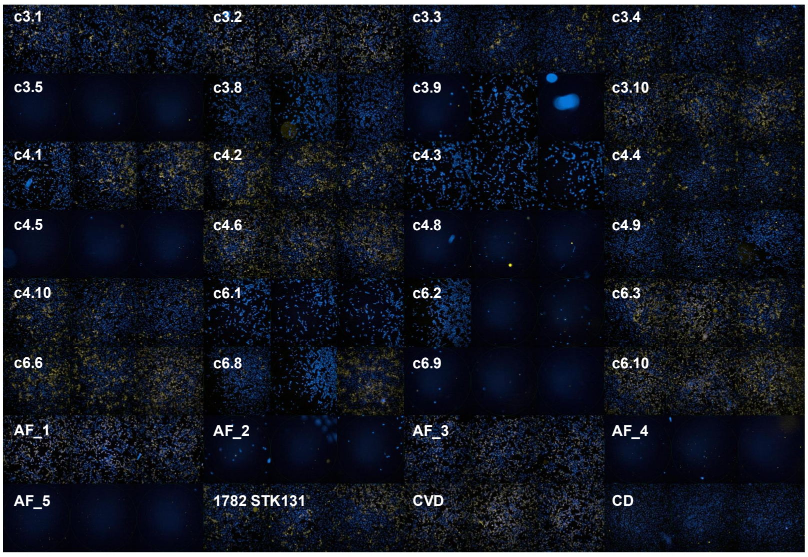

Figure S1-16 Dual-color fluorescent assay screening of unknown compounds as specified, plate 1.
Pictures were taken with ImmunoSpot® Analyzers using Fluoro-X™ FluoroSpot software at 4.48× magnification.

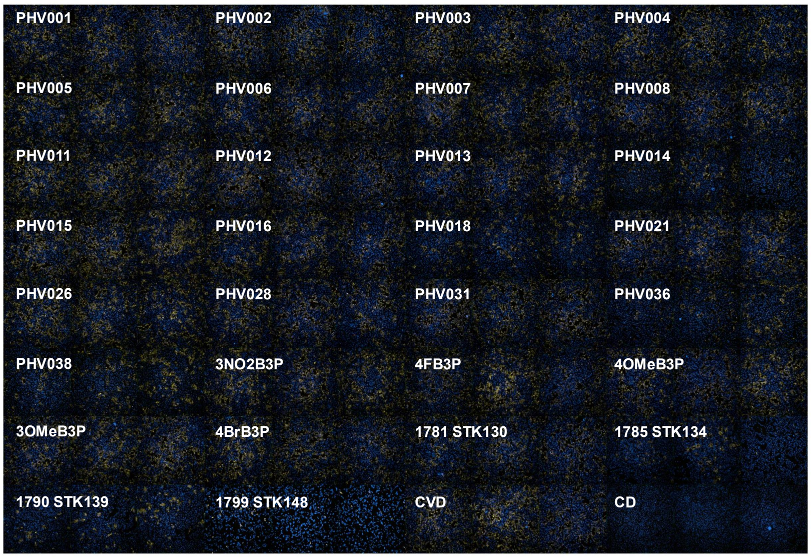

Figure S1-17 Dual-color fluorescent assay screening of unknown compounds as specified, plate 2. Pictures were taken with ImmunoSpot® Analyzers using Fluoro-X™ FluoroSpot software at 4.48× magnification.

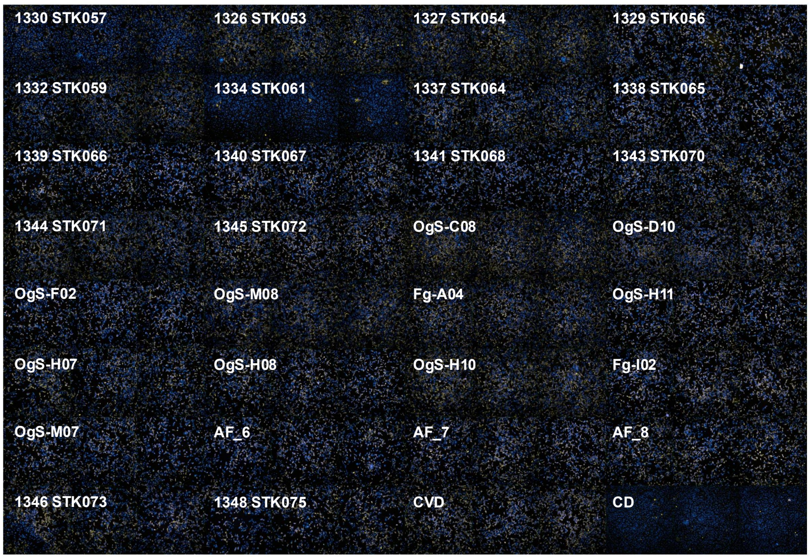

Figure S1-18 Dual-color fluorescent assay screening of unknown compounds as specified, plate 3. Pictures were taken with ImmunoSpot® Analyzers using Fluoro-X™ FluoroSpot software at 4.48× magnification.

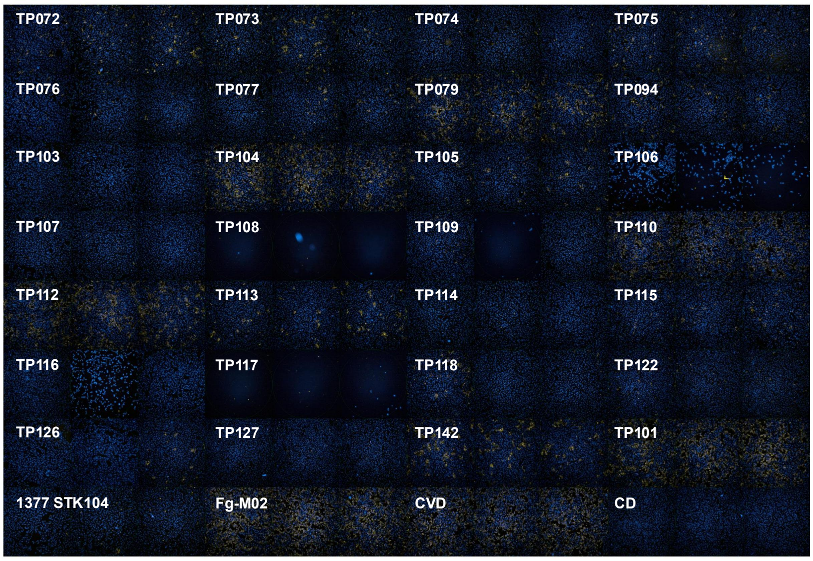

Figure S1-19 Dual-color fluorescent assay screening of unknown compounds as specified, plate 4. Pictures were taken with ImmunoSpot® Analyzers using Fluoro-X™ FluoroSpot software at 4.48× magnification.

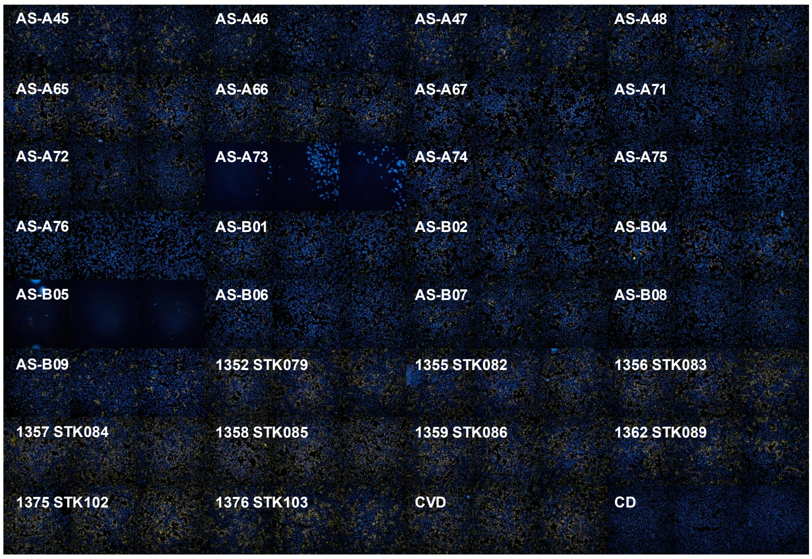

Figure S1-20 Dual-color fluorescent assay screening of unknown compounds as specified, plate 5. Pictures were taken with ImmunoSpot® Analyzers using Fluoro-X™ FluoroSpot software at 4.48× magnification.

Supplementary 2 Raw data and calculation

Table S2-1 Corresponding thresholding range for each well in cell optimization experiment.

Density (cells/well)	Thresholding range											
5×10^3	0-230	0-229	0-228	0-231	0-221	0-231	0-226	0-229	0-231	0-233	0-230	0-231
	0-233	0-231	0-229	0-229	0-230	0-231	0-230	0-230	0-231	0-230	0-230	0-233
1×10^4	0-226	0-225	0-219	0-219	0-225	0-216	0-221	0-219	0-223	0-224	0-228	0-238
	0-228	0-210	0-220	0-221	0-218	0-215	0-219	0-226	0-220	0-224	0-225	0-229
3×10^4	0-161	0-161	0-175	0-161	0-173	0-161	0-161	0-161	0-161	0-161	0-161	0-161
	0-161	0-161	0-161	0-161	0-161	0-161	0-161	0-161	0-161	0-161	0-161	0-161
5×10^4	0-141	0-141	0-141	0-141	0-141	0-141	0-141	0-141	0-141	0-141	0-141	0-141
	0-148	0-138	0-144	0-139	0-135	0-141	0-141	0-141	0-141	0-141	0-141	0-141

Table S2-2 Corresponding coverage area for each well in cell optimization experiment.

Density (cells/well)	Coverage (%)											
5×10^3	82.40	59.48	64.25	65.87	59.41	64.46	62.02	62.61	62.79	63.06	61.93	60.83
	64.71	75.01	62.07	68.96	70.35	63.77	62.99	66.63	59.91	57.93	56.08	52.59
1×10^4	88.60	91.94	87.64	87.54	91.65	85.67	88.98	85.48	85.82	86.74	84.17	87.29
	86.64	84.64	90.17	93.26	87.63	87.20	86.55	90.88	85.63	87.99	82.64	80.00
3×10^4	99.64	99.84	99.74	99.82	99.45	99.80	99.75	99.73	99.60	99.62	99.50	98.60
	99.55	99.72	99.68	99.70	99.76	99.79	99.70	99.70	99.69	99.68	99.56	99.27
5×10^4	99.69	99.71	99.69	99.70	99.72	99.72	99.73	99.68	99.69	99.61	99.68	99.66
	99.70	99.69	99.70	99.66	99.68	99.73	99.74	99.67	99.71	99.70	99.67	99.69

Table S2-3 Summarized coverage area of each density in cell optimization experiment.

Density (cells/well)	Coverage (%)	
	mean	s.d.
5×10^3	63.75	6.08
1×10^4	87.28	3.00
3×10^4	99.62	0.25
5×10^4	99.69	0.03

Table S2-4 Corresponding raw count of total cells for each well in MOI optimization experiment. CD=uninfected control (Cell+DMSO).

Group	Total cells count							
CD	1764	1734	1721	1756	1761	1834	1762	1899
MOI 0.1	1827	1753	1658	1487	1700	1737	1506	1664
MOI 0.3	1303	1095	1235	942	1129	973	970	1190
MOI 0.5	1012	986	1028	1129	1002	907	974	804
MOI 1.0	854	441	450	498	526	486	407	453

Table S2-5 Corresponding percentage of cells left for each well in MOI optimization experiment.

Group	Cells left (%)							
CD	99.16	97.48	96.75	98.71	99.00	103.10	99.05	106.75
MOI 0.1	102.71	98.55	93.20	83.59	95.57	97.65	84.66	93.54
MOI 0.3	73.25	61.56	69.43	52.95	63.47	54.70	54.53	66.90
MOI 0.5	56.89	55.43	57.79	63.47	56.33	50.99	54.75	45.20
MOI 1.0	48.01	24.79	25.30	28.00	29.57	27.32	22.88	25.47

Table S2-6 Corresponding raw count of infected cells for each well in MOI optimization experiment.

Group	Infected cells count							
CD	0	0	0	0	0	0	0	0
MOI 0.1	468	442	512	454	530	380	455	460
MOI 0.3	723	560	660	627	683	527	490	678
MOI 0.5	647	521	519	566	528	485	509	440
MOI 1.0	665	401	395	436	447	413	354	398

Table S2-7 Corresponding inhibition for each well in MOI optimization experiment.

Group	Inhibition (%)							
CD	100.00	100.00	100.00	100.00	100.00	100.00	100.00	100.00
MOI 0.1	-1.16	4.46	-10.67	1.86	-14.56	17.86	1.65	0.57
MOI 0.3	-16.90	9.46	-6.71	-1.37	-10.43	14.79	20.78	-9.62
MOI 0.5	-22.80	1.12	1.49	-7.43	-0.21	7.95	3.39	16.49
MOI 1.0	-51.61	8.58	9.95	0.60	-1.91	5.84	19.29	9.26

Table S2-8 Summarized calculation of each group in MOI optimization experiment.

Group	Cells left (%)		Inhibition (%)		Z’ factor
	mean	s.d.	mean	s.d.	
**CD**	100.00	3.30	100.00	0.00	NA
**MOI 0.1**	93.68	6.63	0.00	9.80	0.706
**MOI 0.3**	62.10	7.55	0.00	13.49	0.595
**MOI 0.5**	55.11	5.31	0.00	11.50	0.655
**MOI 1.0**	28.92	7.99	0.00	21.82	0.345

Table S2-9 Corresponding raw count of infected cells for each well in validation with known compounds. CHX=cycloheximide-treated, CD=uninfected control (Cell+DMSO), ACY=acyclovir-treated, CVD=infected control (Cell+Virus+DMSO).

Group	Infected cells count								
CHX	0	0	0	0	0	0	0	0	0
CD	0	0	0	0	0	0	0	0	0
ACY	382	346	335	417	529	402	369	333	377
CVD	463	412	385	355	467	374	323	348	334

Table S2-10 Corresponding inhibition for each well in validation with known compounds.

Group	Inhibition (%)								
CHX	100.00	100.00	100.00	100.00	100.00	100.00	100.00	100.00	100.00
CD	100.00	100.00	100.00	100.00	100.00	100.00	100.00	100.00	100.00
ACY	0.66	10.03	12.89	-8.44	-37.56	-4.54	4.05	13.41	1.96
CVD	-20.40	-7.14	-0.12	7.69	-21.44	2.74	16.01	9.51	13.15

Table S2-11 Corresponding raw count of total cells for each well in validation with known compounds.

Group	Total cells count								
CHX	1392	1464	1483	1515	1485	1620	1475	1493	1469
CD	1625	1400	1624	1681	1560	1450	1694	1502	1490
ACY	666	734	702	883	952	676	637	558	710
CVD	873	897	1047	787	955	838	682	743	677

Table S2-12 Corresponding percentage of cells left for each well in validation with known compounds.

Group	Cells left (%)								
CHX	89.32	93.94	95.16	97.21	95.29	103.95	94.65	95.80	94.26
CD	104.27	89.83	104.21	107.86	100.10	93.04	108.70	96.38	95.61
ACY	42.73	47.10	45.04	56.66	61.09	43.38	40.87	35.80	45.56
CVD	56.02	57.56	67.18	50.50	61.28	53.77	43.76	47.68	43.44

Table S2-13 Summarized calculation of each group in validation with known compounds.

Group	Inhibition (%)		Cells left (%)	
	mean	s.d.	mean	s.d.
CHX	100.00	0.00	95.51	3.81
CD	100.00	0.00	100.00	6.69
ACY	-0.84	15.67	46.47	7.82
CVD	0	13.75	53.46	7.98

Table S2-14 Corresponding raw count of infected cells for each well in repeatability assessment. R=round of screening

Group	Infected cells count								
	R1			R2			R3		
CHX	0	0	0	0	0	0	0	0	0
CD	0	0	0	0	0	0	0	0	0
ACY	433	332	258	353	346	429	329	297	328
CVD	363	308	469	415	437	387	337	340	347

Table S2-15 Corresponding inhibition for each well repeatability assessment.

Group	Inhibition (%)								
	R1			R2			R3		
CHX	100.00	100.00	100.00	100.00	100.00	100.00	100.00	100.00	100.00
CD	100.00	100.00	100.00	100.00	100.00	100.00	100.00	100.00	100.00
ACY	-13.95	12.63	32.11	14.53	16.22	-3.87	3.61	12.99	3.91
CVD	4.47	18.95	-23.42	-0.48	-5.81	6.30	1.27	0.39	-1.66

Table S2-16 Corresponding raw count of total cells for each well in repeatability assessment.

Group	Total cells count								
	R1			R2			R3		
CHX	1518	1530	1459	1345	1419	1470	1456	1430	1364
CD	1583	1655	1384	1578	1399	1407	1477	1536	1674
ACY	766	557	491	672	689	699	481	583	732
CVD	613	654	1076	676	708	639	526	681	753

Table S2-17 Corresponding percentage of cells left for each well in repeatability assessment.

Group	Cells left (%)								
	R1			R2			R3		
CHX	98.53	99.31	94.70	92.04	97.10	100.59	93.19	91.53	87.31
CD	102.75	107.42	89.83	107.98	95.73	96.28	94.54	98.31	107.15
ACY	49.72	36.15	31.87	45.99	47.15	47.83	30.79	37.32	46.85
CVD	39.79	42.45	69.84	46.26	48.45	43.73	33.67	43.59	48.20

Table S2-18 Summarized calculation of each group in repeatability assessment.

Group	Round	Inhibition (%)		Cells left (%)	
		mean	s.d.	mean	s.d.
CHX	R1	100.00	0.00	97.51	2.47
	R2	100.00	0.00	96.58	4.30
	R3	100.00	0.00	90.68	3.04
CD	R1	100.00	0.00	100.00	9.11
	R2	100.00	0.00	100.00	6.92
	R3	100.00	0.00	100.00	6.47
ACY	R1	10.26	23.12	39.25	9.32
	R2	8.96	11.15	46.99	0.93
	R3	6.84	5.33	38.32	8.08
CVD	R1	0.00	21.54	50.69	16.64
	R2	0.00	6.07	46.14	2.36
	R3	0.00	1.50	41.82	7.42

Table S2-19 Plaque screening results of unknown compounds as specified, plate 1.

Compound	Plaque count			Inhibition (%)		
**c3.1**	11	10	15	99.00	99.09	98.64
**c3.2**	1300	1300	1100	-18.18	-18.18	0.00
**c3.3**	29	17	12	97.36	98.45	98.91
**c3.4**	80	80	50	92.73	92.73	95.45
**c3.5**	5	3	1	99.55	99.73	99.91
**c3.8**	10	12	9	99.09	98.91	99.18
CVD	1000	1400	900	9.09	-27.27	18.18

Table S2-20 Plaque screening results of unknown compounds as specified, plate 2.

Compound	Plaque count			Inhibition (%)		
**c3.9**	8	11	5	99.29	99.03	99.56
**c3.10**	1500	600	800	-32.35	47.06	29.41
**c4.1**	1000	700	1000	11.76	38.24	11.76
**c4.2**	300	300	400	73.53	73.53	64.71
**c4.3**	5	15	11	99.56	98.68	99.03
**c4.4**	50	20	30	95.59	98.24	97.35
CVD	1100	1100	1200	2.94	2.94	-5.88

Table S2-21 Plaque screening results of unknown compounds as specified, plate 3.

Compound	Plaque count			Inhibition (%)		
**c4.5**	3	5	4	99.69	99.48	99.59
**c4.6**	200	600	500	79.31	37.93	48.28
**c4.8**	5	3	4	99.48	99.69	99.59
**c4.9**	30	20	20	96.90	97.93	97.93
**c4.10**	150	30	110	84.48	96.90	88.62
**c6.1**	0	0	0	100.00	100.00	100.00
CVD	400	800	1700	58.62	17.24	-75.86

Table S2-22 Plaque screening results of unknown compounds as specified, plate 4.

Compound	Plaque count			Inhibition (%)		
**c6.2**	7	9	6	99.30	99.10	99.40
**c6.3**	100	300	300	90.00	70.00	70.00
**c6.6**	500	800	500	50.00	20.00	50.00
**c6.8**	20	50	10	98.00	95.00	99.00
**c6.9**	3	2	1	99.70	99.80	99.90
**c6.10**	500	900	800	50.00	10.00	20.00
CVD	1200	1000	800	-20.00	0.00	20.00

Table S2-23 Plaque screening results of unknown compounds as specified, plate 5.

Compound	Plaque count			Inhibition (%)		
AF_1	700	1000	600	48.78	26.83	56.10
AF_2	300	200	300	78.05	85.37	78.05
AF_3	900	1000	800	34.15	26.83	41.46
AF_4	10	20	10	99.27	98.54	99.27
AF_5	30	50	10	97.80	96.34	99.27
1782 STK131	700	800	700	48.78	41.46	48.78
CVD	1100	1600	1400	19.51	-17.07	-2.44

Table S2-24 Plaque screening results of unknown compounds as specified, plate 6.

Compound	Plaque count			Inhibition (%)		
PHV001	500	600	300	46.43	35.71	67.86
PHV002	900	1000	1000	3.57	-7.14	-7.14
PHV003	700	500	600	25.00	46.43	35.71
PHV004	300	400	900	67.86	57.14	3.57
PHV005	900	1300	700	3.57	-39.29	25.00
PHV006	500	1000	800	46.43	-7.14	14.29
CVD	1300	800	700	-39.29	14.29	25.00

Table S2-25 Plaque screening results of unknown compounds as specified, plate 7.

Compound	Plaque count			Inhibition (%)		
**PHV007**	800	1000	900	22.58	3.23	12.90
**PHV008**	900	400	700	12.90	61.29	32.26
**PHV011**	700	900	400	32.26	12.90	61.29
**PHV012**	600	11100	800	41.94	-974.19	22.58
**PHV013**	2300	1500	800	-122.58	-45.16	22.58
**PHV014**	1000	1400	1000	3.23	-35.48	3.23
**CVD**	1200	900	1000	-16.13	12.90	3.23

Table S2-26 Plaque screening results of unknown compounds as specified, plate 8.

Compound	Plaque count			Inhibition (%)		
**PHV015**	900	900	900	77.50	77.50	77.50
**PHV016**	3000	6000	7000	25.00	-50.00	-75.00
**PHV018**	2100	1900	1700	47.50	52.50	57.50
**PHV021**	1200	1500	1500	70.00	62.50	62.50
**PHV026**	1200	900	800	70.00	77.50	80.00
**PHV028**	900	1600	8000	77.50	60.00	-100.00
**CVD**	3000	4000	5000	25.00	0.00	-25.00

Table S2-27 Plaque screening results of unknown compounds as specified, plate 9.

Compound	Plaque count			Inhibition (%)		
**PHV031**	1800	1500	1300	26.03	38.36	46.58
**PHV036**	130	140	130	94.66	94.25	94.66
**PHV038**	1400	1500	1700	42.47	38.36	30.14
**3NO2B3P**	1300	1100	1300	46.58	54.79	46.58
**4FB3P**	1400	1400	1900	42.47	42.47	21.92
**4OMeB3P**	2300	2100	900	5.48	13.70	63.01
**CVD**	2000	2500	2800	17.81	-2.74	-15.07

Table S2-28 Plaque screening results of unknown compounds as specified, plate 10.

Compound	Plaque count			Inhibition (%)		
**3OMeB3P**	1300	500	1000	11.36	65.91	31.82
**4BrB3P**	900	1000	900	38.64	31.82	38.64
**1781 STK130**	600	700	800	59.09	52.27	45.45
**1785 STK134**	400	400	300	72.73	72.73	79.55
**1790 STK139**	140	90	150	90.45	93.86	89.77
**1799 STK148**	70	120	70	95.23	91.82	95.23
**CVD**	1800	1200	1400	-22.73	18.18	4.55

Table S2-29 MTS screening results of unknown compounds as specified, plate 1.

Compound	A490			Viability (%)		
**c3.1**	1.049	1.060	1.062	105.72	106.92	107.15
**c3.2**	1.076	1.095	1.089	108.64	110.80	110.12
**c3.3**	1.084	1.094	1.089	109.58	110.68	110.15
**c3.4**	1.136	1.131	1.124	115.27	114.63	113.90
**c3.5**	0.755	0.641	0.902	73.73	61.28	89.79
**c3.8**	0.944	1.012	0.965	94.33	101.74	96.60
**c3.9**	0.186	0.173	0.812	11.77	10.42	79.93
**c3.10**	1.071	1.055	1.052	108.19	106.38	106.06
**c4.1**	1.050	1.021	1.018	105.87	102.74	102.35
**c4.2**	1.025	1.026	0.999	103.15	103.25	100.30
**c4.3**	0.706	0.758	0.956	68.44	74.03	95.65
**c4.4**	0.981	0.976	0.929	98.38	97.81	92.65
**c4.5**	0.378	0.415	0.522	32.75	36.70	48.36
**c4.6**	1.033	1.044	1.026	104.02	105.25	103.24
**c4.8**	0.333	0.733	0.830	27.81	71.33	81.95
**c4.9**	1.092	1.071	1.088	110.42	108.12	110.03
**c4.10**	0.895	1.021	1.065	88.98	102.72	107.49
**c6.1**	0.444	0.483	0.256	39.89	44.16	19.37
**c6.2**	0.722	0.106	0.829	70.17	3.07	81.85
**c6.3**	1.052	1.073	1.068	106.03	108.39	107.88
**c6.6**	1.097	1.059	1.057	110.99	106.86	106.66
**c6.8**	1.016	1.024	1.009	102.16	103.06	101.37
**c6.9**	0.299	0.653	0.718	24.14	62.59	69.71
**c6.10**	1.013	1.022	1.032	101.89	102.77	103.85
**AF_1**	0.885	1.012	1.019	87.94	101.74	102.44
**AF_2**	0.844	0.831	0.833	83.42	82.03	82.25
**AF_3**	0.991	0.972	1.003	99.41	97.36	100.73
**AF_4**	0.746	0.764	0.639	72.80	74.74	61.17
**AF_5**	0.440	0.701	0.623	39.48	67.89	59.35
**1782 STK131**	1.066	1.082	1.046	107.64	109.37	105.39
**CD**	1.008	0.967	1.013	101.28	96.87	101.85
**blank**	0.079	0.078	0.076	0.12	0.04	-0.16

Table S2-30 MTS screening results of unknown compounds as specified, plate 2.

Compound	A490			Viability (%)		
PHV001	1.037	1.014	1.014	107.35	104.77	104.76
PHV002	1.002	1.033	1.019	103.44	106.90	105.24
PHV003	0.996	1.009	1.000	102.74	104.19	103.21
PHV004	0.974	0.980	0.967	100.29	100.86	99.45
PHV005	1.012	0.985	0.981	104.50	101.44	101.07
PHV006	0.972	0.984	0.963	100.05	101.37	99.01
PHV007	0.987	0.997	1.002	101.65	102.87	103.34
PHV008	0.978	0.974	0.981	100.74	100.21	100.99
PHV011	0.970	0.977	0.988	99.77	100.56	101.79
PHV012	0.976	0.968	0.965	100.52	99.62	99.21
PHV013	0.990	0.978	0.985	102.03	100.64	101.45
PHV014	0.892	0.883	0.906	91.02	90.06	92.62
PHV015	0.981	0.976	0.997	101.08	100.50	102.77
PHV016	0.980	0.987	0.973	100.90	101.70	100.09
PHV018	0.933	0.938	0.937	95.66	96.15	96.05
PHV021	0.997	1.007	0.999	102.83	103.90	103.02
PHV026	0.970	0.962	0.971	99.78	98.93	99.89
PHV028	0.982	0.976	0.983	101.09	100.49	101.20
PHV031	0.993	0.978	0.988	102.37	100.75	101.82
PHV036	0.945	0.956	0.923	96.96	98.17	94.53
PHV038	0.952	0.937	0.947	97.74	96.13	97.22
3NO2B3P	0.989	0.989	0.963	101.94	101.96	98.96
4FB3P	0.961	0.957	0.970	98.74	98.30	99.85
4OMeB3P	0.980	0.980	0.984	100.90	100.89	101.33
3OMeB3P	0.997	0.989	0.983	102.81	101.89	101.22
4BrB3P	0.944	0.937	0.929	96.92	96.04	95.19
1781 STK130	0.845	0.864	0.869	85.74	87.84	88.42
1785 STK134	0.700	0.700	0.751	69.43	69.46	75.18
1790 STK139	1.039	0.989	0.999	107.53	101.93	103.03
1799 STK148	0.907	0.915	1.021	92.68	93.62	105.54
CD	0.973	0.970	0.973	100.11	99.81	100.08
blank	0.080	0.083	0.081	-0.21	0.22	-0.01

Table S2-31 Summarized screening results by standard methods (plaque and MTS assays).

Compounds	Inhibition (%)		Viability (%)	
	mean	s.d.	mean	s.d.
**c3.1**	98.91	0.24	106.60	0.77
**c3.2**	-12.12	10.50	109.86	1.10
**c3.3**	98.24	0.79	110.14	0.55
**c3.4**	93.64	1.57	114.60	0.69
**c3.5**	99.73	0.18	74.93	14.29
**c3.8**	99.06	0.14	97.55	3.80
**c3.9**	99.29	0.26	34.04	39.75
**c3.10**	14.71	41.70	106.88	1.15
**c4.1**	20.59	15.28	103.65	1.93
**c4.2**	70.59	5.09	102.23	1.68
**c4.3**	99.09	0.44	79.37	14.37
**c4.4**	97.06	1.35	96.28	3.16
**c4.5**	99.59	0.10	39.27	8.12
**c4.6**	55.17	21.53	104.17	1.01
**c4.8**	99.59	0.10	60.36	28.69
**c4.9**	97.59	0.60	109.52	1.23
**c4.10**	90.00	6.32	99.73	9.61
**c6.1**	100.00	0.00	34.47	13.25
**c6.2**	99.27	0.15	51.70	42.52
**c6.3**	76.67	11.55	107.43	1.24
**c6.6**	40.00	17.32	108.17	2.45
**c6.8**	97.33	2.08	102.20	0.84
**c6.9**	99.80	0.10	52.15	24.52
**c6.10**	26.67	20.82	102.84	0.98
**AF_1**	43.90	15.23	97.37	8.18
**AF_2**	80.49	4.22	82.57	0.75
**AF_3**	34.15	7.32	99.17	1.69
**AF_4**	99.02	0.42	69.57	7.34
**AF_5**	97.80	1.46	55.57	14.58
**1782 STK131**	46.34	4.22	107.47	2.00

Compounds	Inhibition (%)		Viability (%)	
	mean	s.d.	mean	s.d.
PHV001	50.00	16.37	105.63	1.49
PHV002	-3.57	6.19	105.19	1.73
PHV003	35.71	10.71	103.38	0.74
PHV004	42.86	34.44	100.20	0.71
PHV005	-3.57	32.73	102.34	1.88
PHV006	17.86	26.96	100.14	1.18
PHV007	12.90	9.68	102.62	0.87
PHV008	35.48	24.35	100.65	0.40
PHV011	35.48	24.35	100.71	1.02
PHV012	-303.23	581.16	99.78	0.67
PHV013	-48.39	72.63	101.37	0.70
PHV014	-9.68	22.35	91.23	1.29
PHV015	77.50	0.00	101.45	1.18
PHV016	-33.33	52.04	100.90	0.81
PHV018	52.50	5.00	95.95	0.26
PHV021	65.00	4.33	103.25	0.57
PHV026	75.83	5.20	99.53	0.52
PHV028	12.50	97.82	100.93	0.39
PHV031	36.99	10.34	101.65	0.82
PHV036	94.52	0.24	96.56	1.85
PHV038	36.99	6.28	97.03	0.83
3NO2B3P	49.32	4.75	100.96	1.73
4FB3P	35.62	11.86	98.96	0.80
4OMeB3P	27.40	31.12	101.04	0.25
3OMeB3P	36.36	27.56	101.97	0.80
4BrB3P	36.36	3.94	96.05	0.87
1781 STK130	52.27	6.82	87.33	1.41
1785 STK134	75.00	3.94	71.36	3.31
1790 STK139	91.36	2.19	104.16	2.97
1799 STK148	94.09	1.97	97.28	7.17

Table S2-32 Dual-color fluorescent assay screening results of unknown compounds as specified, plate 1.

Compounds	Infected cells	Inhibition (%)	Total cells	Cells left (%)
c3.1	241	54.15	1067	64.65
	137	73.94	1126	68.23
	41	92.20	901	54.60
c3.2	436	17.06	995	60.29
	489	6.98	1215	73.62
	438	16.68	1142	69.20
c3.3	34	93.53	1352	81.92
	75	85.73	1534	92.95
	135	74.32	1656	100.34
c3.4	146	72.23	1584	95.98
	67	87.25	1484	89.92
	121	76.98	1559	94.47
c3.5	0	100.00	4	0.24
	0	100.00	3	0.18
	0	100.00	0	0.00
c3.8	12	97.72	1043	63.20
	6	98.86	811	49.14
	8	98.48	1270	76.95
c3.9	0	100.00	13	0.79
	5	99.05	340	20.60
	0	100.00	6	0.36
c3.10	360	31.52	1517	91.92
	341	35.13	1643	99.56
	396	24.67	1664	100.83
c4.1	61	88.40	802	48.60
	274	47.88	1332	80.71
	356	32.28	1475	89.38
c4.2	353	32.85	1534	92.95
	299	43.12	1609	97.50
	200	61.95	1549	93.86

Compounds	Infected cells	Inhibition (%)	Total cells	Cells left (%)
**c4.3**	5	99.05	424	25.69
	4	99.24	334	20.24
	9	98.29	208	12.60
**c4.4**	128	75.65	1503	91.07
	83	84.21	1674	101.43
	65	87.63	1581	95.80
**c4.5**	0	100.00	1	0.06
	0	100.00	1	0.06
	2	99.62	8	0.48
**c4.6**	423	19.53	1521	92.16
	432	17.82	1491	90.35
	363	30.94	1461	88.53
**c4.8**	3	99.43	11	0.67
	0	100.00	13	0.79
	2	99.62	7	0.42
**c4.9**	22	95.81	1454	88.10
	34	93.53	1462	88.59
	30	94.29	1184	71.74
**c4.10**	216	58.91	1561	94.59
	121	76.98	1314	79.62
	96	81.74	1488	90.16
**c6.1**	17	96.77	363	22.00
	38	92.77	270	16.36
	7	98.67	285	17.27
**c6.2**	15	97.15	884	53.56
	0	100.00	8	0.48
	0	100.00	18	1.09
**c6.3**	344	34.56	1379	83.56
	539	-2.54	1418	85.92
	309	41.22	1621	98.22
**c6.6**	280	46.73	1521	92.16
	295	43.88	1576	95.50
	479	8.88	1527	92.53

Compounds	Infected cells	Inhibition (%)	Total cells	Cells left (%)
c6.8	25	95.24	1251	75.80
	10	98.10	636	38.54
	284	45.97	1129	68.41
c6.9	0	100.00	5	0.30
	0	100.00	0	0.00
	0	100.00	0	0.00
c6.10	459	12.68	1570	95.13
	484	7.93	1491	90.35
	554	-5.39	1472	89.19
AF_1	450	14.39	833	50.47
	484	7.93	985	59.68
	395	24.86	1124	68.11
AF_2	0	100.00	13	0.79
	0	100.00	0	0.00
	0	100.00	16	0.97
AF_3	304	42.17	1040	63.02
	364	30.75	1102	66.77
	351	33.23	1002	60.72
AF_4	1	99.81	13	0.79
	3	99.43	1	0.06
	0	100.00	0	0.00
AF_5	0	100.00	2	0.12
	0	100.00	0	0.00
	0	100.00	1	0.06
1782 STK131	133	74.70	1256	76.11
	143	72.80	1412	85.56
	320	39.12	1502	91.01
CVD	444	15.54	1258	76.23
	631	-20.04	1219	73.86
	502	4.50	1096	66.41
CD	0	100.00	1688	102.28
	0	100.00	1548	93.80
	0	100.00	1715	103.92

Table S2-33 Dual-color fluorescent assay screening results of unknown compounds as specified, plate 2.

Compounds	Infected cells	Inhibition (%)	Total cells	Cells left (%)
PHV001	397	8.38	1943	104.37
	440	-1.54	1888	101.41
	384	11.38	2044	109.79
PHV002	424	2.15	1949	104.69
	376	13.23	1979	106.30
	292	32.62	1984	106.57
PHV003	473	-9.15	1953	104.91
	414	4.46	1830	98.30
	370	14.62	2016	108.29
PHV004	401	7.46	1942	104.32
	426	1.69	1833	98.46
	299	31.00	1859	99.86
PHV005	395	8.85	1679	90.19
	376	13.23	1934	103.89
	450	-3.85	1722	92.50
PHV006	343	20.85	1536	82.51
	368	15.08	1562	83.90
	306	29.38	1617	86.86
PHV007	400	7.69	1329	71.39
	323	25.46	1436	77.14
	332	23.38	1350	72.52
PHV008	340	21.54	1473	79.12
	330	23.85	1559	83.74
	431	0.54	1988	106.79
PHV011	464	-7.08	1860	99.91
	448	-3.38	1709	91.80
	473	-9.15	1731	92.98
PHV012	411	5.15	1490	80.04
	370	14.62	1583	85.03
	312	28.00	1730	92.93

Compounds	Infected cells	Inhibition (%)	Total cells	Cells left (%)
PHV013	302	30.31	1665	89.44
	311	28.23	1654	88.85
	339	21.77	1349	72.46
PHV014	84	80.62	1285	69.02
	167	61.46	1307	70.21
	58	86.62	1689	90.73
PHV015	525	-21.15	1860	99.91
	492	-13.54	1687	90.62
	430	0.77	1272	68.33
PHV016	276	36.31	1230	66.07
	359	17.15	1367	73.43
	301	30.54	1511	81.16
PHV018	146	66.31	1602	86.05
	145	66.54	1800	96.69
	106	75.54	1183	63.55
PHV021	340	21.54	1540	82.72
	349	19.46	1568	84.23
	359	17.15	2001	107.48
PHV026	471	-8.69	1935	103.94
	417	3.77	1835	98.57
	354	18.31	1303	69.99
PHV028	360	16.92	1329	71.39
	385	11.15	1629	87.50
	307	29.15	1829	98.25
PHV031	361	16.69	1596	85.73
	313	27.77	1564	84.01
	315	27.31	1326	71.23
PHV036	81	81.31	1622	87.13
	104	76.00	1598	85.84
	235	45.77	2000	107.43
PHV038	152	64.92	2049	110.06
	213	50.85	2051	110.17
	261	39.77	1293	69.45

Compounds	Infected cells	Inhibition (%)	Total cells	Cells left (%)
3NO2B3P	368	15.08	1419	76.22
	350	19.23	1338	71.87
	366	15.54	1208	64.89
4FB3P	272	37.23	1271	68.27
	422	2.62	1175	63.12
	164	62.15	1428	76.71
4OMeB3P	391	9.77	1570	84.33
	273	37.00	1406	75.52
	441	-1.77	1916	102.92
3OMeB3P	393	9.31	1772	95.18
	456	-5.23	1822	97.87
	457	-5.46	1644	88.31
4BrB3P	455	-5.00	1715	92.12
	367	15.31	1637	87.93
	329	24.08	1694	90.99
1781 STK130	247	43.00	1762	94.65
	281	35.15	1692	90.89
	287	33.77	1724	92.61
1785 STK134	48	88.92	1704	91.53
	145	66.54	1712	91.96
	10	97.69	1895	101.79
1790 STK139	78	82.00	1933	103.83
	111	74.38	1835	98.57
	160	63.08	2018	108.40
1799 STK148	57	86.85	910	48.88
	42	90.31	751	40.34
	24	94.46	827	44.42
CVD	426	1.69	1890	101.52
	569	-31.31	1867	100.29
	305	29.62	1967	105.66
CD	0	100.00	1870	100.45
	0	100.00	1771	95.13
	0	100.00	1944	104.42

Table S2-34 Dual-color fluorescent assay screening results of unknown compounds as specified, plate 3.

Compounds	Infected cells	Inhibition (%)	Total cells	Cells left (%)
**1330 STK057**	149	73.75	1203	67.21
	152	73.22	1224	68.38
	223	60.72	1421	79.39
**1326 STK053**	445	21.61	1582	88.38
	384	32.35	1589	88.77
	398	29.89	1548	86.48
**1327 STK054**	376	33.76	1476	82.46
	436	23.19	1425	79.61
	423	25.48	1506	84.13
**1329 STK056**	506	10.86	926	51.73
	438	22.84	795	44.41
	425	25.13	787	43.97
**1332 STK059**	266	53.14	1032	57.65
	336	40.81	1370	76.54
	374	34.12	1398	78.10
**1334 STK061**	21	96.30	1328	74.19
	15	97.36	1405	78.49
	39	93.13	1496	83.58
**1337 STK064**	406	28.48	1198	66.93
	457	19.50	1260	70.39
	438	22.84	1366	76.31
**1338 STK065**	517	8.93	942	52.63
	554	2.41	938	52.40
	445	21.61	757	42.29
**1339 STK066**	338	40.46	676	37.77
	380	33.06	723	40.39
	411	27.60	707	39.50
**1340 STK067**	495	12.80	894	49.94
	441	22.31	897	50.11
	511	9.98	1022	57.09

Compounds	Infected cells	Inhibition (%)	Total cells	Cells left (%)
1341 STK068	441	22.31	787	43.97
	470	17.20	822	45.92
	471	17.03	804	44.92
1343 STK070	482	15.09	1046	58.44
	525	7.52	1069	59.72
	441	22.31	966	53.97
1344 STK071	474	16.50	1193	66.65
	470	17.20	1267	70.78
	406	28.48	1245	69.55
1345 STK072	576	-1.47	1061	59.27
	600	-5.70	1049	58.60
	605	-6.58	1095	61.17
OgS-C08	609	-7.28	1562	87.26
	587	-3.41	1480	82.68
	511	9.98	1566	87.49
OgS-D10	499	12.10	1326	74.08
	525	7.52	1349	75.36
	556	2.06	1128	63.02
OgS-F02	375	33.94	727	40.61
	454	20.02	828	46.26
	492	13.33	879	49.11
OgS-M08	461	18.79	1451	81.06
	428	24.60	1399	78.16
	428	24.60	1521	84.97
Fg-A04	607	-6.93	1364	76.20
	562	1.00	1348	75.31
	593	-4.46	1564	87.37
OgS-H11	506	10.86	847	47.32
	546	3.82	812	45.36
	463	18.44	711	39.72
OgS-H07	465	18.09	954	53.30
	429	24.43	994	55.53
	435	23.37	962	53.74

Compounds	Infected cells	Inhibition (%)	Total cells	Cells left (%)
OgS-H08	471	17.03	954	53.30
	520	8.40	936	52.29
	432	23.90	739	41.28
OgS-H10	523	7.87	1380	77.09
	485	14.56	1433	80.06
	539	5.05	1431	79.94
Fg-I02	500	11.92	870	48.60
	605	-6.58	965	53.91
	561	1.17	822	45.92
OgS-M07	404	28.83	729	40.73
	480	15.44	981	54.80
	307	45.92	632	35.31
AF_6	395	30.42	711	39.72
	443	21.96	761	42.51
	398	29.89	719	40.17
AF_7	480	15.44	856	47.82
	545	3.99	1124	62.79
	538	5.23	798	44.58
AF_8	497	12.45	1005	56.15
	473	16.68	814	45.47
	462	18.61	848	47.37
1346 STK073	498	12.27	1100	61.45
	343	39.58	844	47.15
	378	33.41	796	44.47
1348 STK075	289	49.09	592	33.07
	364	35.88	681	38.04
	387	31.83	754	42.12
CVD	573	-0.94	979	54.69
	536	5.58	930	51.96
	594	-4.64	1100	61.45
CD	0	100.00	1699	94.92
	0	100.00	1841	102.85
	0	100.00	1830	102.23

Table S2-35 Dual-color fluorescent assay screening results of unknown compounds as specified, plate 4.

Compounds	Infected cells	Inhibition (%)	Total cells	Cells left (%)
TP072	92	78.20	2163	103.89
	104	75.36	2122	101.92
	141	66.59	2268	108.93
TP073	55	86.97	2201	105.72
	168	60.19	2374	114.02
	49	88.39	2123	101.97
TP074	41	90.28	2123	101.97
	25	94.08	2063	99.09
	12	97.16	2170	104.23
TP075	74	82.46	2426	116.52
	115	72.75	2130	102.31
	54	87.20	2145	103.03
TP076	23	94.55	2003	96.21
	26	93.84	1974	94.81
	18	95.73	2135	102.55
TP077	21	95.02	2030	97.50
	73	82.70	2255	108.31
	45	89.34	2024	97.21
TP079	299	29.15	2196	105.48
	290	31.28	1990	95.58
	241	42.89	2196	105.48
TP094	82	80.57	2298	110.37
	48	88.63	2065	99.18
	43	89.81	2074	99.62
TP103	0	100.00	1994	95.77
	7	98.34	1915	91.98
	5	98.82	2098	100.77
TP104	438	-3.79	2147	103.12
	389	7.82	2038	97.89
	321	23.93	2052	98.56

Compounds	Infected cells	Inhibition (%)	Total cells	Cells left (%)
TP105	48	88.63	2169	104.18
	38	91.00	2107	101.20
	63	85.07	2187	105.04
TP106	1	99.76	655	31.46
	2	99.53	263	12.63
	0	100.00	62	2.98
TP107	4	99.05	1855	89.10
	14	96.68	1968	94.52
	2	99.53	2153	103.41
TP108	0	100.00	1	0.05
	0	100.00	0	0.00
	0	100.00	1	0.05
TP109	27	93.60	2276	109.32
	0		18	
	7	98.34	2074	99.62
TP110	308	27.01	2014	96.73
	330	21.80	1837	88.23
	302	28.44	1916	92.03
TP112	210	50.24	2060	98.94
	349	17.30	2173	104.37
	254	39.81	2142	102.88
TP113	58	86.26	2191	105.24
	42	90.05	2270	109.03
	101	76.07	2169	104.18
TP114	0	100.00	2103	101.01
	2	99.53	2085	100.14
	4	99.05	2105	101.10
TP115	38	91.00	2344	112.58
	43	89.81	2246	107.88
	64	84.83	1906	91.55
TP116	2	99.53	2233	107.25
	13		526	
	0	100.00	1819	87.37

Compounds	Infected cells	Inhibition (%)	Total cells	Cells left (%)
TP117	0	100.00	1	0.05
	0	100.00	0	0.00
	1	99.76	14	0.67
TP118	106	74.88	2146	103.07
	0	100.00	1954	93.85
	14	96.68	2003	96.21
TP122	30	92.89	2512	120.65
	16	96.21	2419	116.19
	25	94.08	2354	113.06
TP126	0	100.00	1758	84.44
	0	100.00	1493	71.71
	48	88.63	2227	106.96
TP127	9	97.87	2410	115.75
	9	97.87	2419	116.19
	8	98.10	2358	113.26
TP142	178	57.82	1762	84.63
	133	68.48	1952	93.76
	88	79.15	1704	81.84
TP101	276	34.60	1953	93.80
	343	18.72	1836	88.18
	404	4.27	1850	88.86
1377 STK104	8	98.10	1600	76.85
	19	95.50	1832	87.99
	18	95.73	1850	88.86
Fg-M02	500	-18.48	1852	88.95
	528	-25.12	1752	84.15
	468	-10.90	1866	89.63
CVD	447	-5.92	1847	88.71
	378	10.43	1866	89.63
	441	-4.50	1786	85.78
CD	0	100.00	2060	98.94
	0	100.00	2105	101.10
	0	100.00	2081	99.95

Table S2-36 Dual-color fluorescent assay screening results of unknown compounds as specified, plate 5.

Compounds	Infected cells	Inhibition (%)	Total cells	Cells left (%)
AS-A45	152	64.98	1774	87.66
	205	52.76	1841	90.97
	208	52.07	1951	96.41
AS-A46	109	74.88	1930	95.37
	49	88.71	1490	73.63
	104	76.04	1760	86.97
AS-A47	230	47.00	1854	91.62
	171	60.60	1814	89.64
	188	56.68	1714	84.70
AS-A48	162	62.67	1523	75.26
	174	59.91	1395	68.93
	92	78.80	1444	71.36
AS-A65	355	18.20	1646	81.34
	385	11.29	1701	84.06
	313	27.88	1634	80.74
AS-A66	249	42.63	1806	89.24
	285	34.33	1747	86.33
	276	36.41	1708	84.40
AS-A67	163	62.44	1259	62.21
	163	62.44	1318	65.13
	141	67.51	1126	55.64
AS-A71	34	92.17	1430	70.66
	29	93.32	1375	67.95
	25	94.24	1393	68.84
AS-A72	109	74.88	1806	89.24
	87	79.95	1850	91.42
	107	75.35	1764	87.17
AS-A73	0	100.00	9	0.44
	0	100.00	105	5.19
	0	100.00	65	3.21

Compounds	Infected cells	Inhibition (%)	Total cells	Cells left (%)
AS-A74	224	48.39	1319	65.18
	176	59.45	1451	71.70
	207	52.30	1479	73.09
AS-A75	3	99.31	1323	65.38
	1	99.77	1213	59.94
	1	99.77	1332	65.82
AS-A76	17	96.08	1049	51.84
	10	97.70	1186	58.61
	11	97.47	1239	61.23
AS-B01	111	74.42	1504	74.32
	86	80.18	1300	64.24
	91	79.03	1311	64.78
AS-B02	158	63.59	1432	70.76
	123	71.66	1409	69.63
	118	72.81	1277	63.10
AS-B04	188	56.68	1508	74.52
	186	57.14	1315	64.98
	225	48.16	1541	76.15
AS-B05	0	100.00	1	0.05
	0	100.00	0	0.00
	0	100.00	3	0.15
AS-B06	63	85.48	1518	75.01
	45	89.63	1456	71.95
	68	84.33	1614	79.76
AS-B07	116	73.27	1884	93.10
	109	74.88	1756	86.77
	93	78.57	1777	87.81
AS-B08	47	89.17	1474	72.84
	24	94.47	1426	70.47
	70	83.87	1646	81.34
AS-B09	109	74.88	1538	76.00
	124	71.43	1703	84.15
	152	64.98	1524	75.31

Compounds	Infected cells	Inhibition (%)	Total cells	Cells left (%)
**1352 STK079**	368	15.21	1881	92.95
	406	6.45	1952	96.46
	304	29.95	1842	91.02
**1355 STK082**	291	32.95	1788	88.35
	334	23.04	1834	90.63
	388	10.60	1690	83.51
**1356 STK083**	365	15.90	1748	86.38
	369	14.98	1679	82.97
	401	7.60	1869	92.36
**1357 STK084**	486	-11.98	1798	88.85
	477	-9.91	1755	86.72
	453	-4.38	1784	88.16
**1358 STK085**	478	-10.14	1619	80.00
	483	-11.29	1738	85.88
	511	-17.74	1651	81.58
**1359 STK086**	453	-4.38	1822	90.03
	395	8.99	1694	83.71
	375	13.59	1753	86.62
**1362 STK089**	333	23.27	1737	85.83
	320	26.27	1878	92.80
	206	52.53	1635	80.79
**1375 STK102**	425	2.07	1585	78.32
	460	-5.99	1720	84.99
	531	-22.35	1561	77.14
**1376 STK103**	368	15.21	1983	97.99
	394	9.22	1948	96.26
	373	14.06	1994	98.53
CVD	446	-2.76	1795	88.70
	442	-1.84	1645	81.29
	414	4.61	1652	81.63
CD	0	100.00	2055	101.55
	0	100.00	1978	97.74
	0	100.00	2038	100.71

Table S2-37 Summarized screening results by dual color fluorescent assay.

Compounds	Inhibition (%)		Cells left (%)	
	mean	s.d.	mean	s.d.
**1782 STK131**	62.21	20.01	84.23	7.54
**1781 STK130**	37.31	4.98	92.71	1.88
**1785 STK134**	84.38	16.07	95.09	5.80
**1790 STK139**	73.15	9.52	103.60	4.92
**1799 STK148**	90.54	3.81	44.55	4.27
**TP072**	73.38	6.05	104.92	3.62
**TP073**	78.52	15.89	107.24	6.17
**TP074**	93.84	3.44	101.76	2.58
**TP075**	80.81	7.37	107.28	8.01
**TP076**	94.71	0.96	97.85	4.12
**TP077**	89.02	6.17	101.01	6.32
**TP079**	34.44	7.40	102.18	5.71
**TP103**	99.05	0.85	96.17	4.41
**TP104**	9.32	13.92	99.86	2.85
**TP105**	88.23	2.98	103.47	2.02
**TP106**	99.76	0.24	15.69	14.49
**TP094**	86.33	5.03	103.06	6.34
**TP115**	88.55	3.27	104.00	11.04
**TP114**	99.53	0.47	100.75	0.53
**TP107**	98.42	1.52	95.68	7.23
**TP109**	95.97	3.35	104.47	6.86
**TP108**	100.00	0.00	0.03	0.03
**TP112**	35.78	16.83	102.07	2.80
**TP113**	84.12	7.23	106.15	2.55
**TP116**	99.76	0.34	97.31	14.06
**TP117**	99.92	0.14	0.24	0.38
**TP118**	90.52	13.65	97.71	4.79
**TP122**	94.39	1.68	116.63	3.81
**TP126**	96.21	6.57	87.70	17.85
**TP127**	97.95	0.14	115.07	1.58

Compounds	Inhibition (%)		Cells left (%)	
	mean	s.d.	mean	s.d.
**TP110**	25.75	3.49	92.33	4.26
**TP142**	68.48	10.66	86.74	6.23
**TP101**	19.19	15.17	90.28	3.07
**c3.1**	73.43	19.03	62.49	7.07
**c3.2**	13.57	5.71	67.70	6.79
**c3.3**	84.53	9.66	91.74	9.27
**c3.4**	78.82	7.68	93.46	3.15
**c3.5**	100.00	0.00	0.14	0.13
**c3.8**	98.35	0.58	63.10	13.91
**c3.9**	99.68	0.55	7.25	11.56
**c3.10**	30.44	5.31	97.43	4.82
**c4.1**	56.18	28.97	72.89	21.48
**c4.2**	45.97	14.76	94.77	2.40
**c4.3**	98.86	0.50	19.51	6.57
**c4.4**	82.50	6.17	96.10	5.19
**c4.5**	99.87	0.22	0.20	0.24
**c4.6**	22.76	7.14	90.35	1.82
**c4.8**	99.68	0.29	0.63	0.19
**c4.9**	94.55	1.16	82.81	9.59
**c4.10**	72.54	12.04	88.12	7.69
**c6.1**	96.07	3.01	18.54	3.03
**c6.2**	99.05	1.65	18.38	30.47
**c6.3**	24.41	23.58	89.23	7.87
**c6.6**	33.16	21.08	93.40	1.83
**c6.8**	79.77	29.30	60.92	19.73
**c6.9**	100.00	0.00	0.10	0.17
**c6.10**	5.07	9.37	91.56	3.15
**AS-A45**	56.61	7.26	91.68	4.42
**AS-A46**	79.88	7.67	85.32	10.96
**AS-A47**	54.76	7.00	88.65	3.56

Compounds	Inhibition (%)		Cells left (%)	
	mean	s.d.	mean	s.d.
AS-A48	67.13	10.20	71.85	3.19
AS-A65	19.12	8.33	82.05	1.77
AS-A66	37.79	4.32	86.66	2.44
AS-A67	64.13	2.93	60.99	4.86
AS-A71	93.24	1.04	69.15	1.39
AS-A72	76.73	2.80	89.28	2.13
AS-A73	100.00	0.00	2.95	2.38
AS-A74	53.38	5.61	69.99	4.22
AS-A75	99.62	0.27	63.71	3.27
AS-A76	97.08	0.87	57.22	4.84
AS-B01	77.88	3.05	67.78	5.67
AS-B02	69.35	5.02	67.83	4.13
AS-B04	53.99	5.06	71.88	6.03
AS-B05	100.00	0.00	0.07	0.08
AS-B06	86.48	2.79	75.57	3.93
AS-B07	75.58	2.72	89.23	3.39
AS-B08	89.17	5.30	74.88	5.72
AS-B09	70.43	5.03	78.49	4.92
1330 STK057	69.23	7.38	71.66	6.72
1326 STK053	27.95	5.63	87.88	1.23
1327 STK054	27.48	5.56	82.07	2.29
1329 STK056	19.61	7.66	46.70	4.36
1332 STK059	42.69	9.65	70.76	11.38
1334 STK061	95.60	2.20	78.75	4.70
1337 STK064	23.61	4.54	71.21	4.75
1338 STK065	10.98	9.76	49.11	5.90
1339 STK066	33.71	6.45	39.22	1.33
1340 STK067	15.03	6.46	52.38	4.08
1341 STK068	18.85	3.00	44.93	0.98
1343 STK070	14.97	7.40	57.37	3.02

Compounds	Inhibition (%)		Cells left (%)	
	mean	s.d.	mean	s.d.
**1344 STK071**	20.73	6.72	68.99	2.12
**1345 STK072**	-4.58	2.73	59.68	1.33
OgS-C08	-0.23	9.06	85.81	2.71
OgS-D10	7.22	5.03	70.82	6.79
OgS-F02	22.43	10.51	45.33	4.32
OgS-M08	22.67	3.36	81.40	3.42
Fg-A04	-3.46	4.06	79.63	6.72
OgS-H11	11.04	7.31	44.13	3.95
OgS-H07	21.96	3.40	54.19	1.18
OgS-H08	16.44	7.77	48.96	6.66
OgS-H10	9.16	4.89	79.03	1.68
Fg-I02	2.17	9.29	49.48	4.07
OgS-M07	30.06	15.28	43.61	10.06
AF_6	27.42	4.74	40.80	1.50
AF_7	8.22	6.29	51.73	9.72
AF_8	15.91	3.15	49.66	5.69
AF_1	15.73	8.54	59.42	8.82
AF_2	100.00	0.00	0.59	0.52
AF_3	35.38	6.00	63.50	3.06
AF_4	99.75	0.29	0.28	0.44
AF_5	100.00	0.00	0.06	0.06
**1346 STK073**	28.42	14.32	51.02	9.13
**1348 STK075**	38.93	9.03	37.75	4.53
**1352 STK079**	17.20	11.88	93.48	2.76
**1355 STK082**	22.20	11.20	87.50	3.63
**1356 STK083**	12.83	4.55	87.23	4.75
**1357 STK084**	-8.76	3.93	87.91	1.08
**1358 STK085**	-13.06	4.10	82.49	3.04
**1359 STK086**	6.07	9.33	86.79	3.17
**1362 STK089**	34.02	16.10	86.48	6.03

Compounds	Inhibition (%)		Cells left (%)	
	mean	s.d.	mean	s.d.
**1375 STK102**	-8.76	12.44	80.15	4.24
**1376 STK103**	12.83	3.18	97.60	1.19
**1377 STK104**	96.45	1.44	84.57	6.70
**Fg-M02**	-18.17	7.11	87.58	2.99
**PHV001**	6.08	6.76	105.19	4.25
**PHV002**	16.00	15.42	105.85	1.02
**PHV003**	3.31	11.93	103.83	5.08
**PHV004**	13.38	15.53	100.88	3.06
**PHV005**	6.08	8.87	95.52	7.33
**PHV006**	21.77	7.20	84.42	2.22
**PHV007**	18.85	9.72	73.68	3.05
**PHV008**	15.31	12.84	89.88	14.82
**PHV011**	-6.54	2.92	94.90	4.38
**PHV012**	15.92	11.48	86.00	6.50
**PHV013**	26.77	4.45	83.58	9.63
**PHV014**	76.23	13.14	76.65	12.20
**PHV015**	-11.31	11.13	86.28	16.23
**PHV016**	28.00	9.83	73.55	7.55
**PHV018**	69.46	5.26	82.09	16.92
**PHV021**	19.38	2.19	91.48	13.88
**PHV026**	4.46	13.51	90.83	18.25
**PHV028**	19.08	9.19	85.71	13.52
**PHV031**	23.92	6.27	80.32	7.92
**PHV036**	67.69	19.17	93.46	12.11
**PHV038**	51.85	12.61	96.56	23.48
**3NO2B3P**	16.62	2.28	70.99	5.72
**4FB3P**	34.00	29.90	69.36	6.86
**4OMeB3P**	15.00	19.91	87.59	13.99
**3OMeB3P**	-0.46	8.46	93.79	4.93
**4BrB3P**	11.46	14.92	90.35	2.17
